# Disorder-tunable entanglement at infinite temperature

**DOI:** 10.1126/sciadv.adj3822

**Published:** 2023-12-22

**Authors:** Hang Dong, Jean-Yves Desaules, Yu Gao, Ning Wang, Zexian Guo, Jiachen Chen, Yiren Zou, Feitong Jin, Xuhao Zhu, Pengfei Zhang, Hekang Li, Zhen Wang, Qiujiang Guo, Junxiang Zhang, Lei Ying, Zlatko Papić

**Affiliations:** ^1^ School of Physics, ZJU-Hangzhou Global Scientific and Technological Innovation Center, and Zhejiang Province Key Laboratory of Quantum Technology and Device, Zhejiang University, Hangzhou 310027, China.; ^2^ School of Physics and Astronomy, University of Leeds, Leeds, UK.

## Abstract

Emerging quantum technologies hold the promise of unravelling difficult problems ranging from condensed matter to high-energy physics while, at the same time, motivating the search for unprecedented phenomena in their setting. Here, we use a custom-built superconducting qubit ladder to realize non-thermalizing states with rich entanglement structures in the middle of the energy spectrum. Despite effectively forming an “infinite” temperature ensemble, these states robustly encode quantum information far from equilibrium, as we demonstrate by measuring the fidelity and entanglement entropy in the quench dynamics of the ladder. Our approach harnesses the recently proposed type of non-ergodic behavior known as “rainbow scar,” which allows us to obtain analytically exact eigenfunctions whose ergodicity-breaking properties can be conveniently controlled by randomizing the couplings of the model without affecting their energy. The on-demand tunability of quantum correlations via disorder allows for in situ control over ergodicity breaking, and it provides a knob for designing exotic many-body states that defy thermalization.

## Teaser

A custom-built superconducting quantum processor creates robust entangled states that can be tuned by disorder.

## INTRODUCTION

The abundance of entanglement and other types of correlations in many-body systems make them an attractive resource for quantum information processing. Quantum coherence, however, is typically fragile, even in systems that can be considered well isolated from the environment: Coherence rapidly deteriorates in the presence of a finite density of quasiparticle excitations above the systems’ ground state. This is a consequence of thermalization—the ultimate fate of generic systems comprising many interacting degrees of freedom (*1*–*3*). If thermalization breaks down, new types of dynamical behavior and phases of matter can emerge. For example, finely tuned one-dimensional systems (*4*) can evade thermalization due to their rich symmetry structure known as quantum integrability (*5*). On the other hand, in real materials, disorder is ubiquitous and, if strong enough, it can strongly suppress thermalization by turning the system into an Anderson insulator (*6*) or its interacting cousin, the many-body localized (MBL) phase (*7*, *8*).

The ability to suppress thermalization while retaining a high degree of control over entanglement is key to robust technological applications based on many-body quantum systems. Integrable systems do not meet this requirement as they are restricted to one spatial dimension and require fine tuning of the parameters. In MBL systems, bulk excitations are localized, regardless of their energy density, which indeed can effectively protect the information stored in the degrees of freedom at the system's boundary (*9*, *10*). Nevertheless, entanglement in MBL states is typically bounded by “area-law” scaling (*11*). This limits their applications in quantum-enhanced metrology, which often rely on large multipartite entanglement (*12*). The latter entanglement structures have recently been identified (*13*, *14*) in a class of systems known as quantum many-body scars (QMBS) (*15*–*17*). When QMBS systems are prepared in special initial states, their dynamics become trapped in a subspace that does not mix with the thermalizing bulk of the spectrum, leading to the coherent time evolution of local observables (*18*–*23*). The observation of QMBS has triggered a flurry of theoretical efforts to understand and classify the general mechanisms of weak ergodicity breaking in isolated quantum systems (*24*–*33*).

In this work, inspired by our state-of-the-art superconducting qubit processor in which the qubit-qubit coupling can be broadly tuned to encompass opposite coupling signs, we demonstrate the existence of entanglement structures that persist far from equilibrium and can be deterministically tuned by disorder. The approach is inspired by the rainbow scar construction (*34*, *35*), which creates Bell pairs between qubits belonging to two halves of the system. We show that our model hosts several distinct families of QMBS states and entanglement structures. While the first family is a direct realization of the rainbow construction, the second family emerges from a hitherto unexplored mechanism: It is obtained by acting on the first family with the Hamiltonian of a single subsystem. By making the couplings spatially inhomogeneous, we can then turn these states into disordered QMBS states whose exact wave functions and entanglement structure can still be written down in analytic form. Unlike their energies, the structure of these exact eigenstates can be explicitly modulated via the disorder profile, allowing the tuning of their properties. We experimentally observe the two types of entanglement via their characteristic ergodicity-breaking signatures in quantum dynamics at late times.

## RESULTS

### The model and its symmetries

**Fig. 1.**
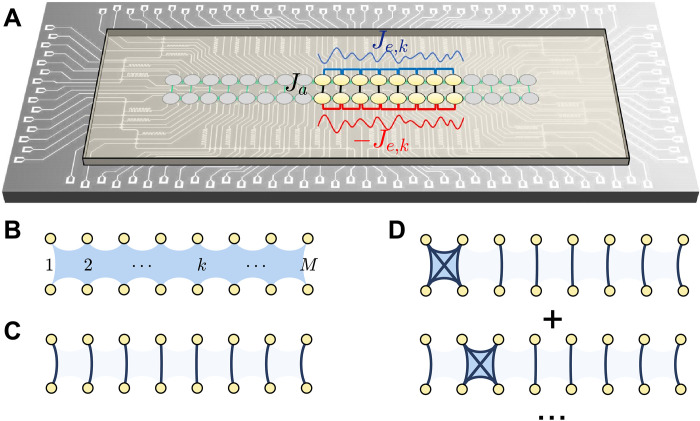
The device and entanglement structures. (**A**) Micrograph of the superconducting quantum processor in a ladder configuration. The tunable couplings, *J_a_* and *J_e_*, between nearest-neighbor qubits, belonging to the same or opposite rows, are indicated. Blue and red curves illustrate the disordered coupling strengths ±*J*_*e*,*k*_, carrying opposite signs in the two rows. (**B** to **D**) Schematic representation of entanglement structure for a thermalizing state, the first family, and second family of scars, respectively. The shaded blue region in (B) indicates large entanglement between all qubits in the thermalizing case. In (C) and (D), dark curves depict the Bell pair entanglement of neighboring qubits, with dimers locally forming ∣**T**〉 states. The tetramer configurations denote doublon-holon entanglement $(|DH\rangle +|HD\rangle )/\sqrt{2}$ , characteristic of the second scar family.

Our superconducting quantum processor contains *N* = 2*M* qubits arranged in a ladder configuration, depicted in Fig. 1A, with two horizontal rungs containing *M* qubits each. The coupling strength between a pair of nearest-neighbor transmon qubits can be capacitively tuned by a coupler, enabling broad ranges of [−8,8] and [−8, −2] MHz for the parallel and vertical couplings, respectively (see the Supplementary Materials for further characteristics of the device). We denote the states of each qubit by ∣∘〉 and ∣•〉. The qubits belonging to the top row are described by ${\hat{u}}^{\alpha }$ Pauli matrices (with α = *x*, *y*, *z*), while ${\hat{d}}^{\alpha }$ are Pauli matrices acting on the bottom-row qubits. The Hamiltonian can be written as

$$\hat{H}={\hat{H}}_{u}⊗1+1⊗{\hat{H}}_{d}+{\hat{H}}_{\mathrm{int}}$$ (1)

where the top/bottom row and inter-row Hamiltonians, respectively, are given by

$${\hat{H}}_{\sigma =u,d}/2\pi =\sum_{k=1}^{M-1}\pm \frac{J_{e,k}}{2}({\hat{\sigma }}_{k}^{x}{\hat{\sigma }}_{k+1}^{x}+{\hat{\sigma }}_{k}^{y}{\hat{\sigma }}_{k+1}^{y})+\sum_{k=1}^{M}\omega_{k}{\hat{\sigma }}_{k}^{z},$$

$${\hat{H}}_{\mathrm{int}}/2\pi =\sum_{k=1}^{M}\frac{J_{a}}{2}({\hat{u}}_{k}^{x}{\hat{d}}_{k}^{x}+{\hat{u}}_{k}^{y}{\hat{d}}_{k}^{y})$$ (2)

Here, the intralayer coupling amplitude *J*_*e*,*k*_ and the frequency ω*_k_* can be site dependent, allowing for the possibility of disorder, while the inter-row coupling *J_a_* is required to be uniform (see the Supplementary Materials). The rainbow construction mandates that the bottom row of qubits must have intra-row coupling amplitudes of opposite sign. One can easily verify that the two Hamiltonians are related by the mirror transformation ${\hat{H}}_{d}=-M{\hat{H}}_{u}^{⋆}M^{†}$, where ℳ simply maps ∣∘〉_*u*,*k*_ ↔ ∣•〉_*d*,*k*_, and ∣•〉_*u*,*k*_ ↔ ∣∘〉_*d,k*_ (see the Supplementary Materials). Crucially, the mirror transformation forces the spectra of ${\hat{H}}_{u}$ and ${\hat{H}}_{d}$ to be identical but of opposite signs.

The model in Eq. 1 has a U(1) symmetry corresponding to the conservation of total magnetization along the *z* direction, and below, unless specified otherwise, we will restrict to its largest sector with zero magnetization or half-filling. The exchange rules of the Hamiltonian give rise to an additional, more subtle, symmetry. Our Hamiltonian can exchange neighboring triplet states, $T\equiv ({}_{∙}^{\circ }{+}_{\circ }^{∙})/\sqrt{2}$, and singlet states, $S\equiv ({}_{∙}^{\circ }{-}_{\circ }^{∙})/\sqrt{2}$. Alternatively, it can create a domain with a doublon $D\equiv ({}_{∙}^{∙})$ or holon $H\equiv ({}_{\circ }^{\circ })$ on each side in which the **T** and **S** are exchanged. Thus, $\hat{H}$ conserves the difference between the number of triplets and singlets, multiplied by a phase factor that counts the number of doublons and holons to the left of a given site (see Materials and Methods for a formal definition of $\hat{Q}$). The $\hat{Q}$ symmetry further splits the half-filling sector of the Hilbert space into *M* + 1 disconnected sectors with quantum numbers −*M*, −*M* + 2, …, *M* − 2, *M*. Working in the largest *Q* sector (at zero magnetization), we checked the statistics of the energy level spacings of $\hat{H}$ using exact diagonalization, finding excellent agreement with the Wigner-Dyson ensemble and very small fluctuations between different disorder realizations (see Materials and Methods), thus indicating that the model is quantum chaotic.

### Two families of rainbow scar entanglements

The rainbow state ∣**I**〉 = ∣**TT**…**T**〉 is an eigenstate of our model in Eq. 1. Two different families of scars can be built from it using operators that commute with the half-system Hamiltonian ${\hat{H}}_{u}$. To construct the first family, we use the operator $\hat{Z}=\sum_{k=1}^{M}{\hat{u}}_{k}^{z}$,which clearly commutes with ${\hat{H}}_{u}$ as the latter conserves *z* magnetization. The powers of $\hat{Z}$ are linearly independent up to ${\hat{Z}}^{M}$; thus, we can apply $\hat{Z}$ up to *M* times. The $\hat{Z}$ operator simply converts triplets into singlets and vice versa. The resulting states ∣*M* − *n*, *n*〉 will be symmetric superpositions of all states with a fixed number *n* of triplets and *M* − *n* singlets. The scarred states of the first family are precisely these states, up to normalization

$$|E_{n}\rangle ={\left(\begin{array}{c} M\\ n \end{array}\right)}^{-1/2}|M-n,n\rangle \propto {\hat{P}}_{2n-M}^{Q}\hat{Z}|E_{n+1}\rangle$$ (3)

where *n* ranges between 0 and *M −* 1 and ∣*E_M_*〉 ≡ ∣**I**〉. The second equality illustrates that we can build *E_n_* recursively from *E*_*n*+1_ by making use of the projector ${\hat{P}}_{q}^{Q}$ on the sector of $\hat{Q}$ with eigenvalue *q*. The projector ${\hat{P}}_{q}^{Q}$ is introduced for convenience as it simplifies the recursion since acting with $\hat{Z}$ on ∣*E_n_*〉 creates a superposition of ∣*E*_*n*−1_〉 and ∣*E*_*n*+1_〉. It can be verified that the state ∣*E_n_*〉 is an eigenstate of $\hat{H}$ with energy *E_n_* = *J_a_*(2*n* − *M*) (see the Supplementary Materials).

While symmetry generators come to mind when we look for operators $\hat{O}$ that commute with ${\hat{H}}_{u}$, we can build a different family of scarred states by using ${\hat{H}}_{u}$ itself as the generator. This gives us the second family of scars

$$|E_{n}^{\prime }\rangle \propto {\hat{P}}_{M-2n}^{Q}\left\{{\hat{H}}_{u}-\left(\sum_{k}\frac{\omega_{k}}{M}\right)\hat{Z}\right\}|E_{n+1}\rangle$$ (4)

with *n* = 1,2, …, *M* − 1. The second term in the square bracket automatically orthogonalizes the states $|E_{n}^{\prime }\rangle$ with respect to the first family of scars. The projectors ${\hat{P}}_{q}^{Q}$ once again isolate $|E_{n-1}^{\prime }\rangle$ from $|E_{n+1}^{\prime }\rangle$. Similar to the first type of scar, the second type of scarred states are also equidistant in energy, occurring at the same energies *E_n_* = *J_a_*(2*n* − *M*) (see the Supplementary Materials).

It is instructive to contrast the two families of scars, Eqs. 3 and 4. While both families occur at the same, regularly spaced energies throughout the spectrum, the total number of states in the second family is smaller by two than in the first family. Furthermore, there are stark differences in entanglement structures. The states belonging to the second family explicitly depend on disorder through their dependence on *J*_*e*,*k*_ and ω*_k_*, unlike the first family. The states in the first family contain only singlets or triplets, with no doublons or holons (Fig. 1C). By contrast, the second family has overlap with states involving a symmetric superposition on a single doublon-holon pair ∣…**DH**…〉 + ∣…**HD**…〉 with weight *J*_*e*,*k*_/2 on top of a background of *M* − *n* − 1 singlets and *n* − 1 triplets (Fig. 1D). They also have overlap with all states with *M* − *n* singlets and *n* triplets, with prefactors depending on the ω*_k_* and on the location of the triplets. The dependence of $|E_{n}^{\prime }\rangle$ states on ω*_k_* and *J*_*e*,*k*_ allows us to tune their properties, such as entanglement entropy or overlap with special initial states.

**Fig. 2.**
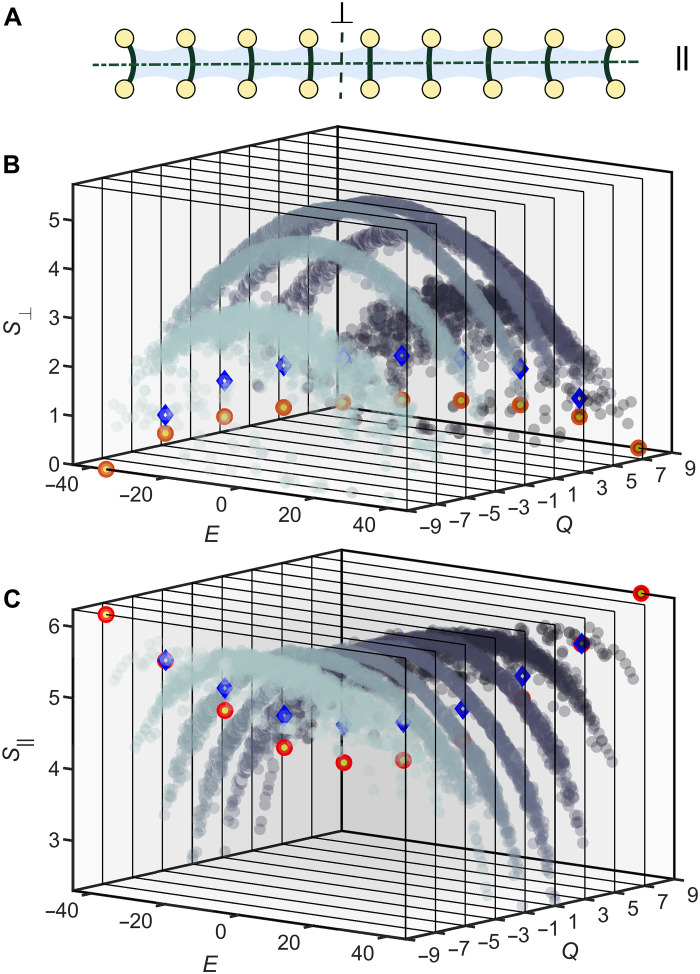
Rainbow entanglement. (**A**) Schematic of the ladder with dashed lines indicating two types of bipartitions. The parallel bipartition splits all entangled pairs in the rainbow state, while the perpendicular bipartition does not split any pair. (**B** and **C**) Bipartite entropy of eigenstates in different *Q*-symmetry sectors for the two cuts shown in (A). The two families of scarred states are highlighted by red circles (first family) and blue diamonds (second family). Their rainbow nature is revealed by the fact that they move between minimal and maximal entropy, depending on the cut. The upper bounds of bipartite entropy in (B) and (C) are given by the Page entropy (2*M* ln 2 − 1)/2 (*48*) and the maximum subsystem entropy *M* ln 2, respectively. Colored cross sections represent different sectors labeled by the values on the *Q* axis. Data are obtained by exact diagonalization with *N* = 18 qubits, *J_a_* = 4, and *J*_*e*,*k*_ ∈ [4, 4.5], ω*_k_* ∈ [0.5, 1.5] drawn from a uniform distribution.

The two families of scars are further identified by their entanglement entropy, *S_A_* = −trρ*_A_* log ρ*_A_*, where ρ*_A_* is the reduced density matrix of the subsystem *A*. The reduced density matrix $\rho_{A}=tr_{\bar{A}}\rho$ is obtained from the full density matrix ρ by tracing out the degrees of freedom belonging to the complement $\bar{A}$ of the subsystem *A*. The rainbow entanglement manifests as a notable difference in entropy depending on the type of bipartition that defines the subsystem *A*, and we will consider two types illustrated in Fig. 2A. For the parallel cut between the two rows of the ladder, i.e., when *A* comprises qubits {*u*_1_, *u*_2_, …, *u_M_*}, the entanglement is large, as the bipartition cuts through an extensive number of Bell pairs. By contrast, for the bipartition perpendicular to the ladder, i.e., when *A* = {*u*_1_, *d*_1_, …, *u*_*M*/2_, *d*_*M*/2_} ≡ {*k* = 1,2, ⋯, *M*/2}, the entanglement is much lower. As seen in Fig. 2 (B and C), this distinction is particularly notable for *E*_0_ and *E_M_* states, which have nearly maximal entropy (i.e., scaling with the number of qubits) in the case of parallel bipartition but low entanglement (i.e., bounded by a constant) in the case of a perpendicular bipartition. Furthermore, for the first type of scarred eigenstate in the middle of the spectrum (*E* = 0), it can be analytically shown that the maximum bipartite entanglement is $S_{1,⊥}^{M\to \infty }=[1+\ln(\pi M/8)]/2$, while for the second type of scarred eigenstates, we numerically established that the disorder allows us to tune the entanglement in the range (*S*_1,⊥_, *S*_1,⊥_ + ln 4) (see the Supplementary Materials).

### Entanglement dynamics

**Fig. 3.**
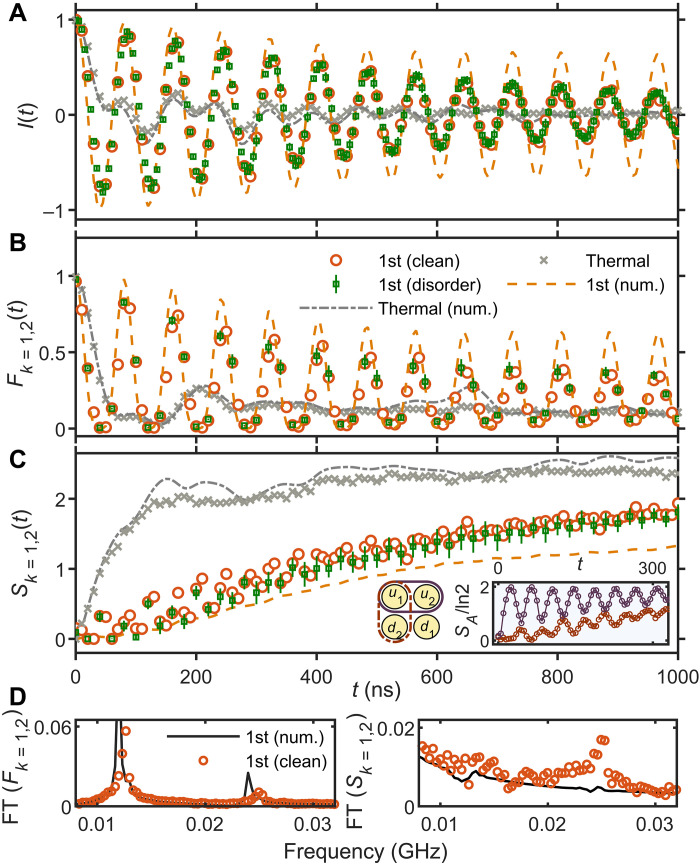
Experimental observation of the dynamical signatures of the first scar family. The ladder with *N* = 10 qubits is initialized in the state ∣Π〉, and the couplings are set to values *J*_*e*,*k*_ = *J_e_* = *J_a_*/3 = −2 MHz. The plots show the measurements of (**A**) population imbalance, (**B**) the four-qubit fidelity, and (**C**) the four-qubit entanglement entropy, specified in the main text. The inset in (C) shows the entropy of a subsystem consisting of two qubits, sketched on the left. Purple dots with error bars stand for the average and standard deviation over eight disorder realizations of *J*_*e*,*k*_, randomly selected from an interval ∈[1,3] MHz. For reference, we also show a typical initial state that thermalizes (see the main text for details). The lines are the results of numerical simulations for the same parameters, including the additional cross couplings *J_x_* ≈ 0.3 MHz and nonlinearity of qubits η ≈ −175 MHz, present in the physical device (see Materials and Methods). The subscripts *k* = 1,2 on the fidelity and entanglement denote the measured subsystem. (**D**) Fourier spectrum of fidelity and entropy dynamics in (B) and (C), respectively.

To observe rainbow entanglements, we use an established diagnostics of QMBS (*18*): the evolution of local observable expectation values in the quench dynamics of the circuit, consisting of two contiguous rows with up to eight qubits each. The structure of the first family of scarred eigenstates implies that they have high overlap with the product state $|\mathrm{II}\rangle =|{}_{\circ }^{∙}{}_{\circ }^{∙}\ldots_{\circ }^{∙}\rangle$. Figure 3A shows the dynamics of population imbalance, $I(t)=(1/N)\sum_{k=1}^{M}\sum_{\sigma =u,d}\langle {\hat{\sigma }}_{k}^{z}(0)\rangle \langle {\hat{\sigma }}_{k}^{z}(t)\rangle .$ The population imbalance in the ∣Π〉 state exhibits remarkable oscillations that persist up to timescales ⁓1 μs. This is in contrast with a typical thermalizing state $|{}_{∙}^{∙}{}_{\circ }^{\circ }{}_{∙}^{∙}{}_{\circ }^{\circ }{}_{\circ }^{∙}\rangle$, for which the population imbalance rapidly decays to zero by ⁓50 ns. A salient feature of the first family of scars is their insensitivity to the disorder in the Hamiltonian couplings. Thus, we expect the coherent dynamics from the ∣Π〉 state to be unchanged when inhomogeneity is introduced in *J*_*e*,*k*_ couplings. This signature is clearly confirmed by experimental observations in Fig. 3A.

Furthermore, we used the quantum tomography technique and obtained the reduced density matrix of the subsystem consisting of qubits *A* = {*k* = 1, 2}, which gives us additional information about the dynamics beyond local observables. The subsystem fidelity, $\sqrt{F_{A}}=\mathrm{tr}\sqrt{\sqrt{\rho_{A}(t)}\rho_{A}(0)\sqrt{\rho_{A}(t)}}$, and entanglement entropy, *S*_*k*=1,2_, are shown in Fig. 3 (B and C). For the initial state ∣Π〉, the subsystem fidelity dynamics undergoes persistent revivals, implying that the initial information is restored many times, with a period of about 80 ns. Meanwhile, for generic initial product states, *F*_*k*=1,2_ quickly decays toward a value close to the inverse of the subsystem Hilbert space dimension, as shown in Fig. 3B. The growth of entanglement entropy also shows a stark contrast between initial states. Compared to the thermal states, the ∣Π〉 state exhibits a slow linear growth with superposed oscillations. The small oscillations are in correspondence with the peaks and valleys observed in the fidelity dynamics, with roughly half the period of the latter, as shown as the Fourier spectrum of the fidelity and entropy dynamics in Fig. 3D. Furthermore, we show the entropy dynamics with different subsystems {*u*_1_, *u*_2_} and {*u*_1_, *d*_1_} in the inset of Fig. 3C, confirming the rainbow entanglement structure previously sketched in Fig. 1C.

We note that the ∣Π〉 initial state can be proven to exhibit perfect revivals and constant-in-time entanglement entropy for our model in Eq. 1 (see the Supplementary Materials). In contrast, Fig. 3 shows a weak population and fidelity decay, along with a slow growth of entanglement. Detailed characterization of the experimental device revealed two extraneous terms not present in the theoretical model, which correspond to diagonal XY couplings and to three-photon occupation not being fully suppressed (see the Supplementary Materials). Numerical simulations, shown by lines in Fig. 3, confirm that these perturbations capture the main sources of decay of local observables and entanglement growth (see Materials and Methods). The effect of these perturbations, however, is sufficiently weak such that clear signatures of the two families of scars can be observed and sharply distinguished, as shown next.

**Fig. 4.**
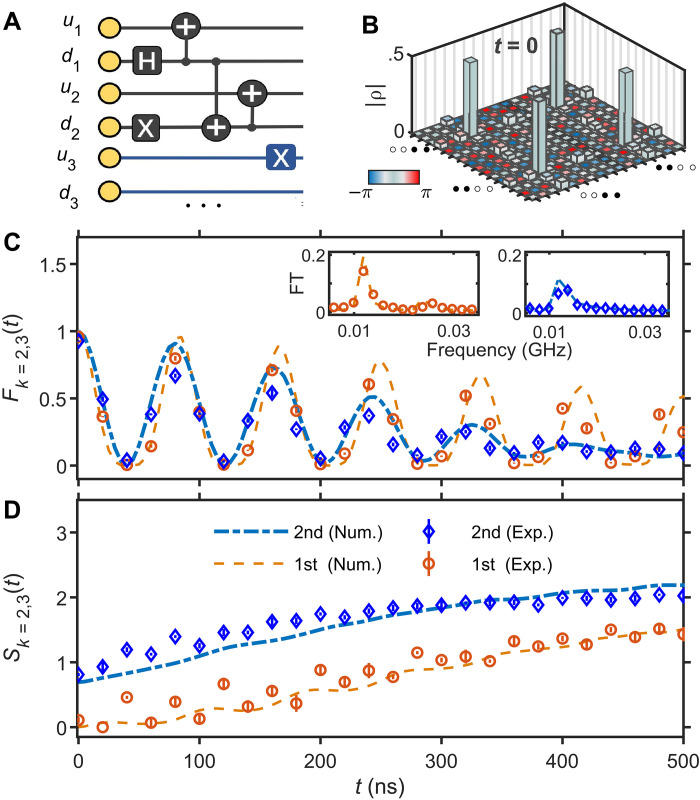
Experimental distinction between the first and second family of scars. (**A**) Circuit diagram for generating the entangled initial state ∣ϕ*_L_*〉 used to probe the dynamics of the second scar family. Symbols “+,” “H,” and “X” stand for CNOT, Hadamard, and Pauli-X gates, respectively. (**B**) Absolute values of the reduced density matrix elements ρ_*k*=1,2_ at *t* = 0, with the color bar denoting their phase. (**C** and **D**) Fidelity and entanglement entropy dynamics of a four-qubit subsystem for initial states ∣Π〉 and ∣ϕ*_L_*〉, which overlap with the first and second family of scars, respectively. Insets of (C) show the Fourier spectra of the fidelity, which distinguish the second family (only one peak) from the first family (two peaks). The superconducting ladder contains *N* = 16 qubits with the same parameters as Fig. 3.

To probe the second scar family, we require a more complicated initial state with an entangled doublon-holon pair: $|\phi_{L}\rangle =\frac{1}{\sqrt{2}}(|{}_{∙}^{∙}{}_{\circ }^{\circ }\rangle +|{}_{\circ }^{\circ }{}_{∙}^{∙}\rangle )⊗|{}_{∙}^{\circ }\ldots {}_{∙}^{\circ }\rangle$, which has predominant overlap with the second family of scarred eigenstates (see the Supplementary Materials). In contrast with the ∣Π〉 state, the state ∣ϕ*_L_*〉 also has a small overlap on the non-scarred subspace; thus, we expect slowly decaying revivals in the latter case, even in the absence of any perturbations. We emphasize that the state ∣ϕ*_L_*〉 is orthogonal to the first family of scars, as the latter do not contain any doublons or holons. To prepare the state ∣ϕ*_L_*〉, we use the circuit scheme in Fig. 4A, which is composed of a few single-qubit and two-qubit gates. By using high-precision tomography measurements, we then obtain the reduced density matrix of the subsystems {*k* = 1,2} or {*k* = 2,3}. The former one at *t* = 0 is visualized in Fig. 4B, demonstrating that the entangled state ∣ϕ*_L_*〉 is successfully prepared.

To reveal the difference between the first and second family of scars, we focus on the subsystem *A*′ ≡ {*k* = 2,3}, whose fidelity *F*_*k*=2,3_(*t*) and entanglement entropy *S*_*k*=2,3_(*t*), are plotted in Fig. 4 (C and D). The subsystem fidelity partially reveals the scarred eigenstates, and the Fourier transformation of *F*_*k*=2,3_ for the state ∣Π〉 has an additional peak compared to the state ∣ϕ*_L_*〉. This difference is related to the fact that the first family of scarred eigenstates contains two more members compared to the second family in Fig. 2B. Since only four qubits {*k* = 2,3} are observed and the coherent information of the rest of the system is traced out, the dynamics of *F*_*k*=2,3_(*t*) approximately reflects the subsystem itself. Thus, the Fourier spectrum of *F*_*k*=2,3_(*t*) involves two peaks and one peak for state ∣Π〉 and ∣ϕ*_L_*〉 due to only two and one dimers, respectively (see the Supplementary Materials). Furthermore, the choice of the subsystem is motivated by the fact that it leads to entropy ln2 for the ∣ϕ*_L_*〉 initial state, while the entropy is still trivially zero for the ∣Π〉 state. This distinction is verified in our experiment, as shown in Fig. 4D.

### Tunable revivals of the second scar family

**Fig. 5.**
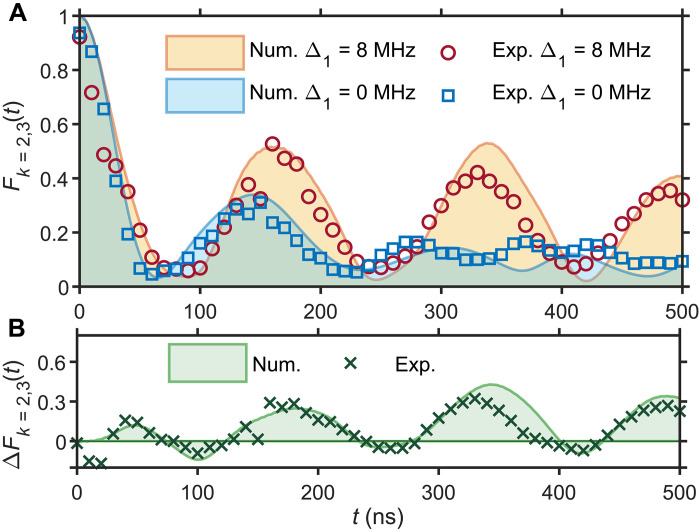
Disorder-tunable scar of the second family. (**A**) Fidelity *F*_*k*=2,3_ for a tunability of Δ_1_ = 0 (blue square) or Δ_1_ = 8 MHz (red circle) over the first inter-dimer coupling *J*_*e*,1_ = *J_e_* + Δ_1_. The experimental data (markers) are for *N* = 8 qubits, *J_a_* = 3.0 MHz, and *J*_*e*,*k*_ ∈ [2.0,3.0] MHz, drawn from a uniform distribution, while the curves are from the numerical simulations based on the experimental model. (**B**) The fidelity difference Δ*F*_*k*=2,3_ between the two cases in (A), illustrating the revival enhancement by Δ_1_. Regions with negative Δ*F*_*k*=2,3_ are due to a slight shift of the revival peaks.

The revivals associated with the second scar family can be conveniently tuned by modulating individual couplings *J*_*e*,*k*_, even in the presence of experimental imperfections. The underlying mechanism behind the revival tunability can be understood by considering the projection of ∣ϕ*_L_*〉 state on the set of scarred eigenstates $|E_{n}^{\prime }\rangle$, which can be shown to be (see the Supplementary Materials)

$$\sum_{n=1}^{M-1}{|\langle \phi_{L}|E_{n}^{\prime }\rangle |}^{2}=\frac{J_{e,1}^{2}}{\sum_{k=1}^{M-1}J_{e,k}^{2}+2\sum_{k=1}^{M}{\bar{\omega }}_{k}^{2}}$$ (5)

with ${\bar{\omega }}_{k}\equiv \omega_{k}-\sum_{j}\omega_{j}/M$. Thus, within the model (Eq. 1), we recover perfect revivals in the limit *J*_*e*,1_ → ∞. To illustrate the *J*_*e*,1_ tunability, in Fig. 5 we measure the fidelity dynamics as *J*_*e*,1_ is modulated by an amount Δ_1_ ∈ [0,8] MHz. Within the accessible range of Δ_1_, the model remains chaotic, yet we observe an increase in fidelity of about 0.3, consistent with the theoretical prediction. This demonstrates that the tunability of the revivals for the second scar family can be achieved with the experimentally relevant values of the parameters.

## DISCUSSION

We have realized multiple families of non-thermalizing states with distinct rainbow entanglement structures throughout the energy spectrum. Our construction allows us to write down exact wave functions for these states, even in the presence of disorder. The existence and stability of scar states in disordered models have recently attracted much attention in theoretical studies (*36*–*40*). A unique aspect of our model is that it allows us to tailor the entanglement of scarred states, while the explicit eigenstate dependence on the disorder profile controls the extent of ergodicity breaking. Signatures of rainbow entanglements were observed by performing quantum state tomography of a many-body state of the ladder following the quench from special initial states, confirming the expected hallmarks of QMBS behavior, such as robust revivals in the fidelity dynamics and slow growth of entanglement far from equilibrium.

While the disorder strength was assumed to be sufficiently weak in this work such that the system overall remains chaotic, the versatility of our setup allows direct access to strong ergodicity-breaking regimes, where many-body localization was recently proposed to give rise to “inverted scarring” phenomena (*41*–*43*). More generally, our work bridges the gap between theoretical studies of QMBS, which place the emphasis on exact constructions of scarred eigenstates (*16*, *17*), and experimental realizations, e.g., in Rydberg atom arrays (*18*, *19*) or optical lattices (*21*), in which the scarred states are not known exactly [apart from a few exceptions (*44*)]. In contrast, our model (Eq. 1) hosts exact scars, while its experimental implementation contains additional perturbations. While these perturbations were shown to be sufficiently weak in our device to allow unambiguous observation of scar signatures even in classical simulations, it would be interesting to study in detail their effects on the stability of QMBS states in larger circuits or higher-dimensional geometries that would rapidly exceed the capability of classical computers.

The flexibility of our construction stems from the fact that the scarred states are not generated by conventional symmetry action but by the Hamiltonian describing one of the rungs of the ladder. For simplicity, we assumed that the latter describes an integrable XY model (although the system overall is non-integrable). This was not essential, however, and our construction can be straightforwardly generalized to cases where the subsystem Hamiltonian is non-integrable (see the Supplementary Materials). The key to implementing the construction was the broad tunability of the experimental device that allowed us to vary the coupling sign, in contrast with traditional multi-qubit superconducting systems (*45*). This tunability offers an additional physical freedom that can be used for designing exotic many-body states that defy quantum thermalization and information scrambling, particularly the creation of multipartite-entangled states without the need for larger spins or complicated interactions (*14*).

## MATERIALS AND METHODS

### Symmetries of the model and statistics of energy levels

**Fig. 6.**
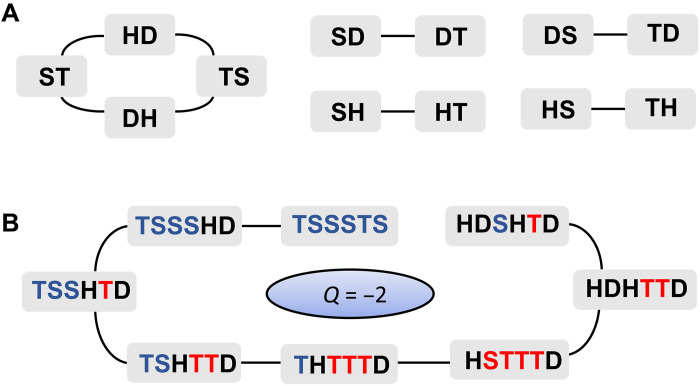
Hamiltonian action and conservation laws. (**A**) Schematic of the Hamiltonian action on neighboring dimers. (**B**) Example of a sequence of states obtained using only allowed processes. The color of the singlet and triplet states indicates if they have been “exchanged” an even (blue) or odd (red) number of times. This exactly corresponds to the parity of the sum of the number of holons and doublons to their left. If one gets +1 for all blue triplets and red singlets, and −1 for all blue singles and red triplets, then for all states in the sequence, the sum is the same and gives *Q* = −2, illustrating the conservation of this charge.

Our model, defined in Eq. 1, conserves the following quantity $\hat{Q}$

$$\hat{Q}=\sum_{k=1}^{M}({\hat{T}}_{k}-{\hat{S}}_{k})\prod_{l=1}^{k-1}{\left(-1\right)}^{{\hat{H}}_{l}+{\hat{D}}_{l}}$$ (6)

where ${\hat{T}}_{k}$, ${\hat{S}}_{k}$, ${\hat{D}}_{k}$, and ${\hat{H}}_{k}$ are projectors on the respective dimer state at the given site *k*. Here, we outline the proof of this statement and provide physical intuition behind the conservation of $\hat{Q}$. We start by considering the action of the Hamiltonian on the dimer basis introduced in "The model and its symmetries." The relevant dynamical terms are summarized in Fig. 6A. Consider a simple configuration like **TSSSTS**. The Hamiltonian rules dictate that we can only exchange **TS** or **ST** into **HD** or **DH**. Therefore, a natural guess for the conserved quantity would be the difference between the total number of triplets and the number of singlets: $\sum_{k}({\hat{T}}_{k}-{\hat{S}}_{k})$. This works perfectly until we start having configurations with neighboring dimers like **SH**. In that case, we can turn it into **HT** and thus change $\sum_{k}({\hat{T}}_{k}-{\hat{S}}_{k})$. However, for that to happen, it means that we first created **HD** and that the dimer that was switched between **S** and **T** must be surrounded by **H** and **D**. Effectively, the Hamiltonian creates domains inside which all **T** and **S** are “exchanged.” Thus, we can keep track of how many times such exchanges have occurred for a given dimer by counting the number of doublons or holons to its left. Figure 6B shows an example of this process.

**Fig. 7.**
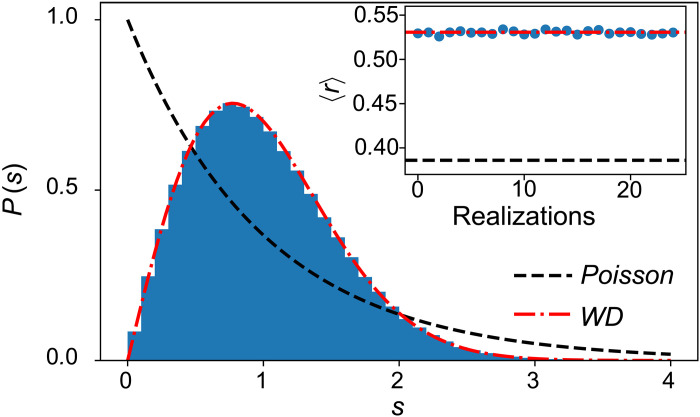
Level statistics. Distribution of energy level spacings *s* ≡ *E*_*n*+1_ − *E_n_* for the model in Eq. 1 with *N* = 20 qubits. Data are for half-filling and *Q* = 0, with 25 disorder realizations. The level statistics displays excellent agreement with the Wigner-Dyson ensemble. The inset shows the average consecutive spacing ratio 〈*r*〉 for each realization, which is very close to the expected value of 0.53 for a chaotic system. Data are for *J_a_* = 3 and *J*_*e*,*k*_ ∈ [2,2.5], ω*_k_* ∈ [0.5,1.5] drawn from a uniform distribution.

While we focused on the half-filling sector in the main text, we note that $\hat{Q}$ is a symmetry at any filling (see the Supplementary Materials for a detailed discussion). The interplay of $\hat{Q}$ and filling leads to a large number of disconnected sectors. Some of them are of small dimension and similar to the ones studied in (*46*). Nonetheless, for large sectors, we find good agreement with the eigenstate thermalization hypothesis predictions, as shown in Fig. 7.

Last, beyond the symmetry $\hat{Q}$, at half-filling the Hamiltonian anticommutes with

$$\hat{C}={\hat{P}}_{d↔u}\prod_{k=1}^{M}{\hat{u}}_{k}^{x}{\hat{d}}_{k}^{x}{\hat{d}}_{k}^{z}$$ (7)

where ${\hat{P}}_{d↔u}$ is an operator exchanging the top and bottom rows. $\hat{C}$ is related to a particle-hole transformation along with a swap between the top and bottom row and an additional phase. In the dimer picture, $\hat{C}$ simply switches **T** and **S** as well as **D** and **H**, with a (−1) phase for every **D** present. Note that, as we are at half-filling, there is always the same number of **D** and **H**, so $\hat{C}={\hat{C}}^{†}$ and ${\hat{C}}^{2}=1$. As it also anti-commutes with $\hat{Q}$, $\hat{C}$ exchanges the sectors with *Q* = *q* and *Q* = −*q*. For *M*-even, we have a sector with *q* = 0; in that sector, the spectrum is symmetric around *E* = 0 because of $\hat{C}$; however, this is not the case in other sectors.

### Experimental protocols

In the main text, we used a quench protocol to observe the many-body scar on the superconducting quantum processor. This includes three main steps: state preparation, interaction, and measurement. In the experimental sequence, we first initialize all the qubits, *Q_i_*, in their ground state at their idle frequency ω*_j_*, the vertical couplers at around their sweet spots, and the horizontal couplers at around the frequency where the coupling strength between two nearest qubits are zero. The initial states are prepared by alternating the single-qubit gates and the two-qubit controlled-π (CZ) gates. The preparation of the first family of scarred state ∣Π〉, which is a product state, is realized by applying single-qubit XY rotations to every qubit. The preparation of the second family of scarred state ∣ϕ*_L_*〉 differs from the first family, as it is a “cat” state. We alternate single- and three two-qubit gates on the former four qubits and apply XY rotations on the other qubits (as illustrated in Fig. 4), with each coupler dynamically switched between nearly off and on to realize the CZ gate. CNOT gates in the experiment circuits are realized by CZ gate with Hadamard gates operated on the target qubit before and after.

In the interaction step, we bias all qubits on resonance at the interaction frequency ω*_i_*. Meanwhile, the couplers are also tuned to turn on the net couplings between neighboring qubits. After waiting for an interaction time *t*, we finally tune all qubit frequencies away from the interacting regime to measure the relevant quantities at their readout frequencies. Repeating this process with varying *t* allows us to obtain the dynamics of the system. In addition, using the quantum state tomography technique, we obtain the reduced density matrix of the subsystem *A*.

**Fig. 8.**
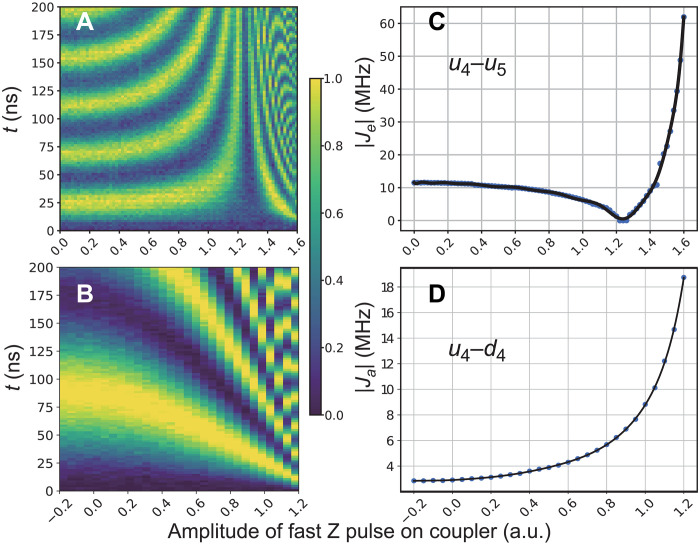
Effective tunable couplings in the experimental device. Swapping dynamics as tuned by the coupler for (**A**) the two adjacent horizontal qubits (*u*_4_ − *u*_5_) and (**B**) the longitudinal qubits (*u*_4_ − *u*_4_). The absolute effective coupling strengths as a function of the coupler Z pulse amplitude [in arbitrary units (a.u.)] are obtained by fitting the oscillations. The horizontal coupling strength (**C**) can be adjusted from positive to negative values, while the longitudinal coupling strength (**D**) can only be tuned in the negative range.

In the experiment, tuning the coupling strengths of all couplers and tuning all qubits on resonance are two very important steps, for which we have conducted careful calibration following the procedure below:

1. Coarse-tune coupling strengths: The coupling strength between each pair of neighboring qubits is coarsely calibrated as a function of the amplitude of the coupler Z pulse, as shown in Fig. 8. In this process, we excite one of the qubits and then place them at ω*_i_* for 200 ns, during which other qubits are placed 50 MHz above ω*_i_*, and other couplers are placed at around their maximum frequency. The coupling strength can be estimated by fitting the swapping dynamics. Following this process, we obtain the functions of all couplers.

2. Fine-tune coupling strengths: Keeping the frequencies of other qubits as above, we apply the corresponding Z pulse on other couplers according to the above results to roughly achieve the designed coupling strength. Then, we slightly change the coupler Z pulse to fine-tune the coupling strength, validated by the swapping dynamics. This process is conducted for each pair of neighboring qubits.

3. Fine-tune frequencies of qubits: The frequencies of qubits may be affected by some factors such as Z pulse distortion, so we adopt the following strategies to minimize this uncertainty, during which all couplers are placed at their desired frequencies. We first apply π/2 pulse on one qubit, then place other qubits at a frequency above ω*_i_* and tune this qubit to ω*_i_* for a fixed delay, and finally measure its accumulated phase ϕ_+_ = Δω_+_ × *t* by tomographic operations. We also measure its accumulated phase ϕ_−_ = Δω_−_ × *t* when other qubits are placed 50 MHz below ω*_i_*. We then slightly change the qubit frequency to make ϕ_+_ + ϕ_−_~0. This process is implemented for every qubit.

### Stability against perturbations

The Hamiltonian describing our experimental device can be written as

$$\begin{array}{c} {\hat{H}}_{\exp}=\hat{H}+{\hat{H}}_{x}+\hat{V},\\ {\hat{H}}_{x}/2\pi =J_{x}\sum_{K=1}^{M-1}({\hat{u}}_{k}^{+}{\hat{d}}_{k+1}^{-}+{\hat{d}}_{k}^{+}{\hat{u}}_{k+1}^{-}+h.c.)\\ \hat{V}/2\pi =\frac{\eta }{2}\sum_{k=1}^{M}({\hat{u}}_{k}^{+}{\hat{u}}_{k}^{+}{\hat{u}}_{k}^{-}{\hat{u}}_{k}^{-}+{\hat{d}}_{k}^{+}{\hat{d}}_{k}^{+}{\hat{d}}_{k}^{-}{\hat{d}}_{k}^{-}) \end{array}$$ (8)

Here, $\hat{H}$ denotes the Hamiltonian of Eq. 1 in the main text, which has been reformulated in terms of bosons, where the standard raising and lowering operators are given by ${\hat{u}}^{\pm }=({\hat{u}}^{x}\pm i{\hat{u}}^{y})/2$ (and, similarly, for ${\hat{d}}^{\pm }$). We consider a maximum of two photons per site. The last two terms represent the experimental imperfections due to the cross coupling *J_x_* between the diagonal qubits and nonlinearity η of the transmon qubit (*22*). The precise values of the *J_x_* couplings as measured in our device are listed in the Supplementary Materials. The terms in Eq. 8 have been included in the numerical simulations presented in Figs. 3 and 4.

**Fig. 9.**
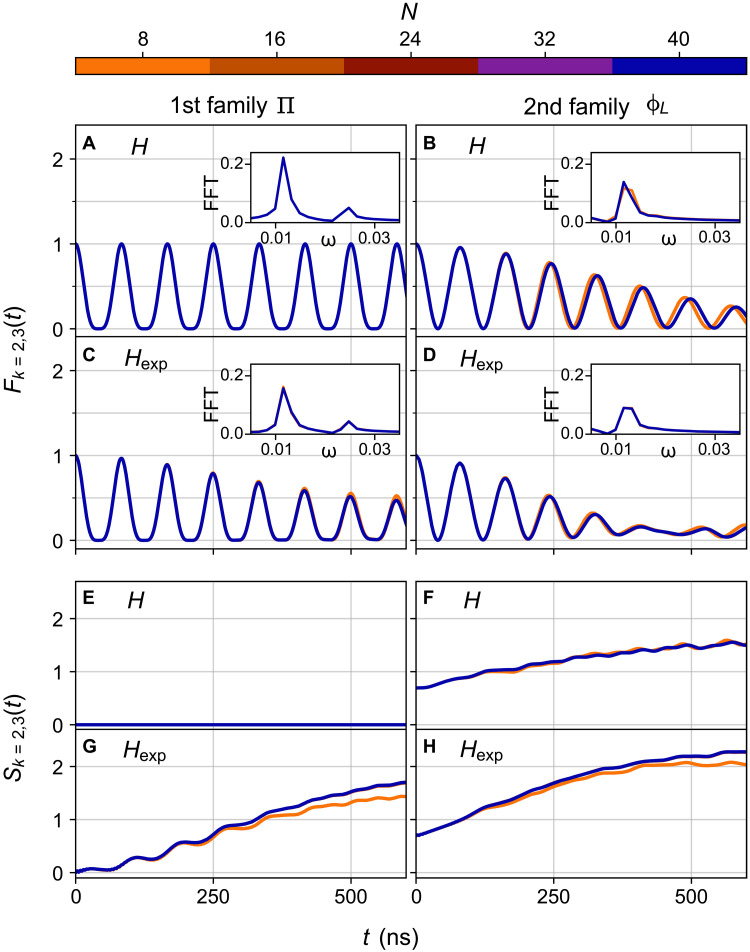
Influence of experimental perturbations. Fidelity and entanglement entropy dynamics of the subsystem {*k* = 2,3} obtained by the numerical simulations of the ideal Hamiltonian $\hat{H}$ in the main text (**A**, **B**, **E**, and **F**) and the Hamiltonian ${\hat{H}}_{\exp}$, which contains the perturbations in Eq. 8 (**C**, **D**, **G**, and **H**). Data for *N* ≥ 24 are obtained using time-dependent variational principle. The coupling parameters are identical to those in Fig. 4 in the main text and are *J_a_* = −6 MHz, *J*_*e*,*k*_ = −2 MHz, η = 175 MHz, and *J_x_* ∈ [0.05,0.45] MHz. Insets show the Fast Fourier Transform (FFT) of the data in the main panels.

The existence of ${\hat{H}}_{x}$ and $\hat{V}$ terms weakens the amplitude of revival dynamics of both families of scar states. However, the perturbed model ${\hat{H}}_{\exp}$ still supports the two scar families and allows us to clearly distinguish between them. In Fig. 9, we demonstrate the stability of our results with respect to these experimental perturbations in large systems *N* ≤ 40 using matrix-product state methods and time-dependent variational principle, as implemented in TenPy libraries (*47*). The accuracy of this calculation is controlled by the bond dimension of the matrix product states, and the convergence was ensured by requiring that the relative error in *F*_*k*=2,3_(*t*) and in *S*_*k*=2,3_(*t*) observables are always below 10^−3^ when comparing the two largest bond dimensions used. Global quantities, such as many-body fidelity and bipartite entanglement entropy, were also monitored and showed good agreement between different bond dimensions. The strength of the cross couplings *J_x_* beyond the 16 first qubits have been randomly drawn from a uniform distribution in [0.05,0.45] MHz and are then kept identical across all system sizes. Consistent with the results in Fig. 4, the fidelity dynamics of subsystem *A* = {*k* = 2,3} involves two frequencies for the first family of scar, one more than in the second family. The initial entanglement entropy is ln2 for the second family of scar, further distinguishing it from the first scar family, which has zero initial value for the entropy. The data in Fig. 9 reveal a remarkably fast convergence in system size. While the smallest system *N* = 8 deviates slightly from larger sizes, we can see that *F*_2,3_(*t*) and *S*_2,3_(*t*) are already fully converged across the experimentally accessible time interval of 500 ns for system sizes *N* ≥ 16.

## Supplementary Materials

### This PDF file includes:

Supplementary Text Figs. S1 to S12 Tables S1 and S2 References

## References

[1] J. M. Deutsch, Quantum statistical mechanics in a closed system. Phys. Rev. A 43, 2046–2049 (1991). 9905246 10.1103/physreva.43.2046

[2] M. Srednicki, Chaos and quantum thermalization. Phys. Rev. E. 50, 888–901 (1994). 10.1103/physreve.50.888 9962049

[3] M. Rigol, V. Dunjko, M. Olshanii, Thermalization and its mechanism for generic isolated quantum systems. Nature 452, 854–858 (2008). 18421349 10.1038/nature06838

[4] T. Kinoshita, T. Wenger, D. S. Weiss, A quantum Newton’s cradle. Nature 440, 900–903 (2006). 16612376 10.1038/nature04693

[5] B. Sutherland, *Beautiful Models: 70 Years of Exactly Solved Quantum Many-body Problems* (World Scientific, 2004).

[6] P. W. Anderson, Absence of diffusion in certain random lattices. Phys. Rev. 109, 1492–1505 (1958).

[7] R. Nandkishore, D. A. Huse, Many-body localization and thermalization in quantum statistical mechanics. Annu. Rev. Condens. Matter. Phys. 6, 15–38 (2015).

[8] D. A. Abanin, E. Altman, I. Bloch, M. Serbyn, Colloquium: Many-body localization, thermalization, and entanglement. Rev. Mod. Phys. 91, 021001 (2019).

[9] D. A. Huse, R. Nandkishore, V. Oganesyan, A. Pal, S. L. Sondhi, Localization-protected quantum order. Phys. Rev. B 88, 014206 (2013).

[10] Y. Bahri, R. Vosk, E. Altman, A. Vishwanath, Localization and topology protected quantum coherence at the edge of hot matter. Nat. Commun. 6, 7341 (2015). 26159426 10.1038/ncomms8341

[11] B. Bauer, C. Nayak, Area laws in a many-body localized state and its implications for topological order. J. Stat. Mech. Theory Exp. 2013, P09005 (2013).

[12] L. Pezzè, A. Smerzi, M. K. Oberthaler, R. Schmied, P. Treutlein, Quantum metrology with nonclassical states of atomic ensembles. Rev. Mod. Phys. 90, 035005 (2018).

[13] J.-Y. Desaules, F. Pietracaprina, Z. Papić, J. Goold, S. Pappalardi, Extensive multipartite entanglement from su(2) quantum many-body scars. Phys. Rev. Lett. 129, 020601 (2022). 35867451 10.1103/PhysRevLett.129.020601

[14] S. Dooley, S. Pappalardi, J. Goold, Entanglement enhanced metrology with quantum many-body scars. Phys. Rev. B 107, 035123 (2023).

[15] M. Serbyn, D. A. Abanin, Z. Papić, Quantum many-body scars and weak breaking of ergodicity. Nat. Phys. 17, 675–685 (2021).

[16] S. Moudgalya, B. A. Bernevig, N. Regnault, Quantum many-body scars and Hilbert space fragmentation: A review of exact results. Rep. Prog. Phys. 85, 086501 (2022). 10.1088/1361-6633/ac73a0 35617909

[17] A. Chandran, T. Iadecola, V. Khemani, R. Moessner, Quantum many-body scars: A quasiparticle perspective. Annu. Rev. Condens. Matter. Phys. 14, 443–469 (2023).

[18] H. Bernien, S. Schwartz, A. Keesling, H. Levine, A. Omran, H. Pichler, S. Choi, A. S. Zibrov, M. Endres, M. Greiner, V. Vuletic, M. D. Lukin, Probing many-body dynamics on a 51-atom quantum simulator. Nature 551, 579–584 (2017). 29189778 10.1038/nature24622

[19] D. Bluvstein, A. Omran, H. Levine, A. Keesling, G. Semeghini, S. Ebadi, T. T. Wang, A. A. Michailidis, N. Maskara, W. W. Ho, S. Choi, M. Serbyn, M. Greiner, V. Vuletić, M. D. Lukin, Controlling quantum many-body dynamics in driven Rydberg atom arrays. Science 371, 1355–1359 (2021). 33632894 10.1126/science.abg2530

[20] P. N. Jepsen, Y. K. E. Lee, H. Lin, I. Dimitrova, Y. Margalit, W. W. Ho, W. Ketterle, Long-lived phantom helix states in Heisenberg quantum magnets. Nat. Phys. 18, 899–904 (2022).

[21] G.-X. Su, H. Sun, A. Hudomal, J.-Y. Desaules, Z.-Y. Zhou, B. Yang, J. C. Halimeh, Z.-S. Yuan, Z. Papić, J.-W. Pan, Observation of many-body scarring in a Bose-Hubbard quantum simulator. Phys. Rev. Res. 5, 023010 (2023).

[22] P. Zhang, H. Dong, Y. Gao, L. Zhao, J. Hao, J.-Y. Desaules, Q. Guo, J. Chen, J. Deng, B. Liu, W. Ren, Y. Yao, X. Zhang, S. Xu, K. Wang, F. Jin, X. Zhu, B. Zhang, H. Li, C. Song, Z. Wang, F. Liu, Z. Papić, L. Ying, H. Wang, Y.-C. Lai, Many-body Hilbert space scarring on a superconducting processor. Nat. Phys. 19, 120–125 (2023).

[23] Y. Yao, L. Xiang, Z. Guo, Z. Bao, Y.-F. Yang, Z. Song, H. Shi, X. Zhu, F. Jin, J. Chen, S. Xu, Z. Zhu, F. Shen, N. Wang, C. Zhang, Y. Wu, Y. Zou, P. Zhang, H. Li, Z. Wang, C. Song, C. Cheng, R. Mondaini, H. Wang, J. Q. You, S.-Y. Zhu, L. Ying, Q. Guo, Observation of many-body Fock space dynamics in two dimensions. Nat. Phys. 19, 1459–1465 (2023).

[24] N. Shiraishi, T. Mori, Systematic construction of counterexamples to the Eigenstate thermalization hypothesis. Phys. Rev. Lett. 119, 030601 (2017). 28777645 10.1103/PhysRevLett.119.030601

[25] S. Moudgalya, N. Regnault, B. A. Bernevig, Entanglement of exact excited states of Affleck-Kennedy-Lieb-Tasaki models: Exact results, many-body scars, and violation of the strong Eigenstate thermalization hypothesis. Phys. Rev. B 98, 235156 (2018).

[26] C. J. Turner, A. A. Michailidis, D. A. Abanin, M. Serbyn, Z. Papić, Weak ergodicity breaking from quantum many-body scars. Nat. Phys. 14, 745–749 (2018).

[27] W. W. Ho, S. Choi, H. Pichler, M. D. Lukin, Periodic orbits, entanglement, and quantum many-body scars in constrained models: Matrix product state approach. Phys. Rev. Lett. 122, 040603 (2019). 30768339 10.1103/PhysRevLett.122.040603

[28] M. Schecter, T. Iadecola, Weak ergodicity breaking and quantum many-body scars in spin-1 XY magnets. Phys. Rev. Lett. 123, 147201 (2019). 31702215 10.1103/PhysRevLett.123.147201

[29] D. K. Mark, C.-J. Lin, O. I. Motrunich, Unified structure for exact towers of scar states in the Affleck-Kennedy-Lieb-Tasaki and other models. Phys. Rev. B 101, 195131 (2020).

[30] N. O’Dea, F. Burnell, A. Chandran, V. Khemani, From tunnels to towers: Quantum scars from Lie algebras and q-deformed Lie algebras. Phys. Rev. Research 2, 043305 (2020).

[31] K. Pakrouski, P. N. Pallegar, F. K. Popov, I. R. Klebanov, Many-body scars as a group invariant sector of Hilbert space. Phys. Rev. Lett. 125, 230602 (2020). 33337167 10.1103/PhysRevLett.125.230602

[32] S. Moudgalya, O. I. Motrunich, Exhaustive characterization of quantum many-body scars using commutant algebras. arXiv:2209.03377 [cond-mat.str-el] (2022).

[33] B. Buča, Unified theory of local quantum many-body dynamics: Eigenoperator thermalization theorems. Phys. Rev. X 13, 031013 (2023).

[34] C. M. Langlett, Z.-C. Yang, J. Wildeboer, A. V. Gorshkov, T. Iadecola, S. Xu, Rainbow scars: From area to volume law. Phys. Rev. B 105, L060301 (2022).

[35] J. Wildeboer, C. M. Langlett, Z.-C. Yang, A. V. Gorshkov, T. Iadecola, S. Xu, Quantum many-body scars from Einstein-Podolsky-Rosen states in bilayer systems. Phys. Rev. B 106, 205142 (2022).

[36] N. Shibata, N. Yoshioka, H. Katsura, Onsager’s scars in disordered spin chains. Phys. Rev. Lett. 124, 180604 (2020). 32441961 10.1103/PhysRevLett.124.180604

[37] I. Mondragon-Shem, M. G. Vavilov, I. Martin, Fate of quantum many-body scars in the presence of disorder. PRX Quantum 2, 030349 (2021).

[38] K. Huang, Y. Wang, X. Li, Stability of scar states in the two-dimensional PXP model against random disorder. Phys. Rev. B 104, 214305 (2021).

[39] B. van Voorden, M. Marcuzzi, K. Schoutens, J. Minář, Disorder enhanced quantum many-body scars in Hilbert hypercubes. Phys. Rev. B 103, L220301 (2021).

[40] G. Zhang, Z. Song, Quantum scars in spin- 1/2 isotropic Heisenberg clusters. New J. Phys. 25, 053025 (2023).

[41] N. S. Srivatsa, H. Yarloo, R. Moessner, A. E. B. Nielsen, Mobility edges through inverted quantum many-body scarring. Phys. Rev. B 108, L100202 (2023).

[42] Q. Chen, Z. Zhu, Inverting multiple quantum many-body scars via disorder. arXiv:2301.03405 [cond-mat.dis-nn] (2023).

[43] M. Iversen, A. E. B. Nielsen, Tower of quantum scars in a partially many-body localized system. Phys. Rev. B 107, 205140 (2023).

[44] C.-J. Lin, O. I. Motrunich, Exact quantum many-body scar states in the Rydberg-blockaded atom chain. Phys. Rev. Lett. 122, 173401 (2019). 31107057 10.1103/PhysRevLett.122.173401

[45] F. Arute, K. Arya, R. Babbush, D. Bacon, J. C. Bardin, R. Barends, R. Biswas, S. Boixo, F. G. S. L. Brandao, D. A. Buell, B. Burkett, Y. Chen, Z. Chen, B. Chiaro, R. Collins, W. Courtney, A. Dunsworth, E. Farhi, B. Foxen, A. Fowler, C. Gidney, M. Giustina, R. Graff, K. Guerin, S. Habegger, M. P. Harrigan, M. J. Hartmann, A. Ho, M. Hoffmann, T. Huang, T. S. Humble, S. V. Isakov, E. Jeffrey, Z. Jiang, D. Kafri, K. Kechedzhi, J. Kelly, P. V. Klimov, S. Knysh, A. Korotkov, F. Kostritsa, D. Landhuis, M. Lindmark, E. Lucero, D. Lyakh, S. Mandrà, J. R. Mc Clean, M. M. Ewen, A. Megrant, X. Mi, K. Michielsen, M. Mohseni, J. Mutus, O. Naaman, M. Neeley, C. Neill, M. Y. Niu, E. Ostby, A. Petukhov, J. C. Platt, C. Quintana, E. G. Rieffel, P. Roushan, N. C. Rubin, D. Sank, K. J. Satzinger, V. Smelyanskiy, K. J. Sung, M. D. Trevithick, A. Vainsencher, B. Villalonga, T. White, Z. J. Yao, P. Yeh, A. Zalcman, H. Neven, J. M. Martinis, Quantum supremacy using a programmable superconducting processor. Nature 574, 505–510 (2019). 31645734 10.1038/s41586-019-1666-5

[46] T. Iadecola, M. Žnidarič, Exact localized and ballistic eigenstates in disordered chaotic spin ladders and the Fermi-Hubbard model. Phys. Rev. Lett. 123, 036403 (2019). 31386440 10.1103/PhysRevLett.123.036403

[47] J. Hauschild, F. Pollmann, Efficient numerical simulations with tensor networks: Tensor network Python (TeNPy). SciPost Phys. Lect., 5 (2018).

[48] D. N. Page, Average entropy of a subsystem. Phys. Rev. Lett. 71, 1291–1294 (1993). 10055503 10.1103/PhysRevLett.71.1291

[49] X. Zhang, W. Jiang, J. Deng, K. Wang, J. Chen, P. Zhang, W. Ren, H. Dong, S. Xu, Y. Gao, F. Jin, X. Zhu, Q. Guo, H. Li, C. Song, A. V. Gorshkov, T. Iadecola, F. Liu, Z. X. Gong, Z. Wang, D. L. Deng, H. Wang, Digital quantum simulation of Floquet symmetry-protected topological phases. Nature 607, 468–473 (2022). 35859194 10.1038/s41586-022-04854-3 PMC9300455

