# Supplementary material for: Disorder-tunable entanglement at infinite temperature
Source: Sci Adv. 2023 Dec 22;9(51):eadj3822. doi: 10.1126/sciadv.adj3822 (PMC10745696; doi:10.1126/sciadv.adj3822)
Supplement: Supplementary file 1 — Supplementary Text Figs. S1 to S12 Tables S1 and S2 References [file sciadv.adj3822_sm.pdf]

Science Advances
AAAS

Supplementary Materials for

Disorder-tunable entanglement at infinite temperature

Hang Dong *et al.*

Corresponding author: Zhen Wang, 2010wangzhen@zju.edu.cn; Lei Ying, leiying@zju.edu.cn;
Zlatko Papić, z.papic@leeds.ac.uk

Sci. Adv. **9**, eadj3822 (2023)
DOI: 10.1126/sciadv.adj3822

This PDF file includes:

Supplementary Text
Tables S1 to S12
Figs. S1 and S2
References

1 Device information

Our experiment is performed on a flip-chip superconducting quantum processor hosting 2×20 frequency-tunable transmon qubits in a ladder configuration. Each pair of adjacent qubits (both in rungs and side rails) are coupled by a tunable transmon coupler. All the control lines and readout resonators are located on a silicon substrate (bottom chip) and all the qubits and couplers are located on a sapphire substrate (top chip). The substrates are connected together with indium bumps as described in Ref. (*49*). Each qubit can be controlled by three control lines. The microwave (XY) line can control the state of the qubit, while the flux (DC/Z) lines can change the frequency of the qubit. The maximum resonance frequencies for the qubits are around 4.6 GHz and can be effectively tuned to below 4.0 GHz. The qubit can be measured using a capacitively coupled readout resonator, with the frequency of the resonator ranging from 6.5 GHz to 6.7 GHz. Each coupler is equipped with its own flux (DC/Z) lines to tune its frequency, with a designed maximum frequency of around 9 GHz. The total coupling strength of each pair of adjacent qubits is composed of direct coupling between them and indirect coupling through the coupler. The former is dominated by the direct capacitance between the qubits while the latter is determined by the capacitance between the qubit and the coupler. Both have been carefully designed so as to realize different tunable ranges in the rungs and side rails. Detailed information about the processor can be found in Tab. S1, including the idle frequencies, average single-qubit gate error, energy relaxation times, and dephasing times. Note that in the experiment, the qubits form a coupled system that is insensitive to each qubit's flux noise. Thus effective dephasing times are usually much longer than the Ramsey dephasing time T_2^*. For example, using the spin echo technique, the dephasing time is typically measured to be around 10 μs. Finally, the estimated values of the J_x cross couplings are provided in Tab. S2.

Table S1: **Parameters of the processor.** ω_j^0 is the maximum frequency of Q_j, known as the sweet spot; ω_j^{i} is the idle frequency of Q_j where single-qubit XY rotations are applied and the average single-qubit gate Pauli error e_{sq} is measured via simultaneous cross entropy benchmarking (XEB). All qubits are biased to $\omega^{\mathrm{I}} \approx 4.375$ GHz to activate the interaction, where the energy relaxation time $T_{1,j}$ and the Ramsey dephasing time $T_{2,j}^*$ of each qubit Q_j are measured.

Qubit	ω_j^0 (GHz)	ω_j^{i} (GHz)	e_{sq} (%)	$T_{1,j}$ (μs)	$T_{2,j}^*$ (μs)
u_1	4.650	4.357	0.532	46.3	1.29
d_1	4.578	3.982	0.442	24.0	1.37
u_2	4.650	4.074	0.619	20.6	1.39
d_2	4.670	4.304	0.620	26.5	0.77
u_3	4.684	4.344	0.718	25.5	1.09
d_3	4.639	3.996	0.647	30.7	1.21
u_4	4.745	4.068	0.973	32.2	0.97
d_4	4.619	4.309	0.589	17.4	1.58
u_5	4.684	4.347	1.456	25.8	0.89
d_5	4.622	4.029	0.760	25.3	1.30
u_6	4.596	4.044	0.635	33.1	2.00
d_6	4.460	4.334	0.475	23.6	1.05
u_7	4.541	4.314	0.976	26.6	1.72
d_7	-	4.064	1.412	15.9	0.84
u_8	-	4.022	0.684	13.6	2.35
d_8	4.569	4.349	0.451	38.6	1.59
Ave.	-	-	0.749	26.6	1.34

2 Finite-size scaling

In Fig. S1 we show the experimental results of imbalance, subsystem fidelity, and entanglement entropy of the first four qubits in system sizes ranging from $N = 10$ to 16 qubits obtained using exact diagonalization. The experimental results are in good agreement with the numerical simulations, complementing similar analysis in the Methods section.

3 Subsystem fidelity for two families of scars

In general, the fidelity of a subsystem A for both pure and mixed initial states is given by

$$F_A(t) = \left(\mathrm{tr}\sqrt{\sqrt{\rho_A(t)}\rho_A(0)\sqrt{\rho_A(t)}}\right)^2 . \tag{S1}$$

Table S2: **Cross coupling strengths.** List of the cross couplings J_x from experimental measurements.

Pairs	J_x (MHz)	Pairs	J_x (MHz)
$u_1 - d_2$	0.19	$d_1 - u_2$	0.10
$u_2 - d_3$	0.30	$d_2 - u_3$	0.34
$u_3 - d_4$	0.37	$d_3 - u_4$	0.45
$u_5 - d_5$	0.31	$d_4 - u_5$	0.33
$u_6 - d_6$	0.25	$d_5 - u_6$	0.26
$u_7 - d_7$	0.19	$d_6 - u_7$	0.18
$u_8 - d_8$	0.28	$d_7 - u_8$	0.16

The reduced density matrix is given by

$$\begin{aligned}\rho_A(t) &= \mathrm{tr}_B \rho(t) = \mathrm{tr}_B \Big(\sum_{n,n'} c_n(t) c^*_{n'}(t) |n\rangle\langle n'| \Big) \\ &= \sum_{\beta,n,n'} \Big(c_n(t) c^*_{n'}(t) \langle b_\beta | b_n\rangle \langle b_{n'} | b_\beta\rangle \Big) |a_n\rangle\langle a_{n'}|,\end{aligned} \tag{S2}$$

where $A = \{a_1, \cdots, a_\alpha, \cdots\}$ and $B = \{b_1, \cdots, b_\beta, \cdots\}$ represent the subsystems A and B, respectively. Here, $\alpha, \beta = 1, \cdots, D_{A,B}$ and $n, n' = 1, \cdots, D$ denote the indices of subsystems and the entire system, respectively, and D, $D_{A,B}$ are the dimensions of entire Hilbert space and subspace A and B.

The first scar family can be probed by studying the dynamics of $|\Pi\rangle$, which is constrained to a hypercube subspace with a single particle in each dimer $\{u_k, d_k\}$. Then, the dynamics of the reduced density matrix is simplified to

$$\begin{aligned}\rho_A(\Pi; t) &= \sum_{\beta,n,n' \in \mathrm{S}} \Big(c_n(t) c^*_{n'}(t) \langle b_\beta | b_n\rangle \langle b_{n'} | b_\beta\rangle \Big) |a_n\rangle\langle a_{n'}| \\ &= 2^{N_B/2} \sum_{\alpha,\alpha' \in \mathrm{S_A}} c_\alpha(t) c^*_{\alpha'}(t) |a_\alpha\rangle\langle a_{\alpha'}|.\end{aligned} \tag{S3}$$

Thus, the reduced density matrix of the subsystem A for $|\Pi\rangle$ is equivalent to an isolated system of size N_A. For the subsystem $A = \{k = 1\}$, its fidelity is a cosine function with only one peak in its Fourier spectrum. As the subsystem size increases, the revival fidelity consists of multiple frequencies in general. This result is confirmed by numerical simulations, as shown in Fig S2.

For the initial state $|\phi_L\rangle$, the fidelity revivals are not exact even for the ideal Hamiltonian $\hat{H}$. In this case, the reduced matrix cannot be simplified as $\rho_A(\Pi; t)$ above. However, to understand the fidelity dynamics it is helpful to consider a small system. For $A = \{k = 1\}$ and $\{k = 1, 2\}$, the corresponding initial state are $|\phi_L\rangle = \left(|\substack{\bullet \\ \bullet}\rangle + |\substack{\circ \\ \circ}\rangle\right)/\sqrt{2}$ and $\left(|\substack{\bullet\circ \\ \bullet\circ}\rangle + |\substack{\circ\bullet \\ \circ\bullet}\rangle\right)/\sqrt{2}$, which are entangled states. Their fidelity dynamics have no revivals. If the subsystem A involves the third dimers, i.e. $A = \{k = 1, 2, 3\}$, the initial state $|\phi_L\rangle = \left(1/\sqrt{2}\right)\left(|\substack{\bullet\circ \\ \bullet\circ}\rangle + |\substack{\circ\bullet \\ \circ\bullet}\rangle\right) \otimes |\substack{\bullet \\ \circ}\rangle$ contains a $|\substack{\bullet \\ \circ}\rangle$. Its fidelity dynamics have one-frequency revival dynamics, as shown in Fig. S3.

Furthermore, we emphasize that the experimental perturbations do not affect our distinction between states Π and ϕ_L. In Figs. S2 and S3, we numerically compute the fidelity dynamics for different subsystem sizes based on the experimental Hamiltonian. The signatures that distinguish the first and second families of scars can be clearly observed.

4 Hamiltonian in the dimer basis

The Hamiltonian from the main text can be re-written as

$$\hat{H} = \sum_{k=1}^{M-1} \hat{h}^{\parallel}_{k,k+1} + \sum_{k=1}^{M} \hat{h}^{\perp}_{k} \tag{S4}$$

with

$$\begin{aligned} \hat{h}^{\parallel}_{k,k+1} &= \frac{J_{e,k}}{2}\left(\hat{\mathbf{u}}^x_k\hat{\mathbf{u}}^x_{k+1} + \hat{\mathbf{u}}^y_k\hat{\mathbf{u}}^y_{k+1} - \hat{\mathbf{d}}^x_k\hat{\mathbf{d}}^x_{k+1} - \hat{\mathbf{d}}^y_k\hat{\mathbf{d}}^y_{k+1}\right), \\ \hat{h}^{\perp}_{k} &= \frac{J_a}{2}\left(\hat{\mathbf{u}}^x_k\hat{\mathbf{d}}^x_k + \hat{\mathbf{u}}^y_k\hat{\mathbf{d}}^y_k\right) + \omega_k\left(\hat{\mathbf{u}}^z_k + \hat{\mathbf{d}}^z_k\right). \end{aligned} \tag{S5}$$

The $\hat{\mathbf{u}}^\alpha$ and $\hat{\mathbf{d}}^\alpha$ are Pauli matrices acting on the top and bottom rows, respectively. The state $\bullet$ denotes an up-spin and $\circ$ denotes a down-spin, such that $\hat{\mathbf{u}}\,|\circ\rangle = -\,|\circ\rangle$ and $\hat{\mathbf{u}}\,|\bullet\rangle = |\bullet\rangle$. The effect of applying the Hamiltonian on any state in the computational (Z) basis is straightforward. However, its effect on the dimer basis is not obvious and we explicitly work it out in this section. To achieve this, it will be convenient to use the above decomposition of the Hamiltonian as a

sum of terms that either act on a single dimer or on two dimers at once. The former corresponds to diagonal terms and hopping perpendicular to the ladder (denoted by $\hat{h}_k^{\perp}$), while the latter encompasses hopping parallel to the ladder and is denoted by $\hat{h}_{k,k+1}^{\parallel}$.

In the main text, we introduced the convenient local dimer basis, spanned by doublon $|\mathbf{D}\rangle$, holon $|\mathbf{H}\rangle$, triplet $|\mathbf{T}\rangle$ and singlet $|\mathbf{S}\rangle$ states. Those were given by:

$$\begin{aligned} |\mathbf{D}\rangle &:= |\substack{\bullet \\ \bullet}\rangle\,, \quad |\mathbf{H}\rangle := |\substack{\circ \\ \circ}\rangle\,, \\ |\mathbf{T}\rangle &:= \frac{1}{\sqrt{2}}\left(|\substack{\circ \\ \bullet}\rangle + |\substack{\bullet \\ \circ}\rangle\right), \\ |\mathbf{S}\rangle &:= \frac{1}{\sqrt{2}}\left(|\substack{\circ \\ \bullet}\rangle - |\substack{\bullet \\ \circ}\rangle\right). \end{aligned} \tag{S6}$$

It is straightforward to see that every state in the dimer basis is an eigenstate of $\hat{h}_k^{\perp}$ with

$$\begin{aligned} \hat{h}_k^{\perp}|\mathbf{H}\rangle &= -2\omega_k|\mathbf{H}\rangle\,, \\ \hat{h}_k^{\perp}|\mathbf{T}\rangle &= \ \ J_a|\mathbf{T}\rangle\,, \\ \hat{h}_k^{\perp}|\mathbf{D}\rangle &= 2\omega_k|\mathbf{D}\rangle\,, \\ \hat{h}_k^{\perp}|\mathbf{S}\rangle &= -J_a|\mathbf{S}\rangle\,. \end{aligned} \tag{S7}$$

The action of $\hat{h}_{k,k+1}^{\parallel}$ is more complicated and requires looking at two neighboring dimers. It is straightforward to show $\hat{h}_{k,k+1}^{\parallel}|\sigma\sigma\rangle = 0$ for any $\sigma = \mathbf{H}, \mathbf{D}, \mathbf{T}, \mathbf{S}$. The non-zero terms are

$$\begin{aligned} \hat{h}_{k,k+1}^{\parallel}|\mathbf{TS}\rangle &= J_e(|\mathbf{HD}\rangle - |\mathbf{DH}\rangle), \\ \hat{h}_{k,k+1}^{\parallel}|\mathbf{TH}\rangle &= \ -J_e|\mathbf{HS}\rangle\,, \\ \hat{h}_{k,k+1}^{\parallel}|\mathbf{SH}\rangle &= \ -J_e|\mathbf{HT}\rangle\,, \\ \hat{h}_{k,k+1}^{\parallel}|\mathbf{TD}\rangle &= J_e|\mathbf{DS}\rangle\,, \\ \hat{h}_{k,k+1}^{\parallel}|\mathbf{SD}\rangle &= J_e|\mathbf{DT}\rangle\,, \\ \hat{h}_{k,k+1}^{\parallel}|\mathbf{HD}\rangle &= J_e(|\mathbf{TS}\rangle - |\mathbf{ST}\rangle). \end{aligned} \tag{S8}$$

The missing combinations can be obtained by flipping the two sites under consideration on the right- and left-hand sides. Importantly, this leads to the following combinations of states being

annihilated by $\hat{h}^{\parallel}_{k,k+1}$:

$$\begin{aligned}\hat{h}^{\parallel}_{k,k+1}\left(|\mathbf{TS}\rangle+|\mathbf{ST}\rangle\right)&=0,\\ \hat{h}^{\parallel}_{k,k+1}\left(|\mathbf{HD}\rangle+|\mathbf{DH}\rangle\right)&=0.\end{aligned} \tag{S9}$$

5 Structure of scarred eigenstates

The rainbow scar construction (*33, 34*) is based on two subsystems (labeled "1" and "2"), with respective Hamiltonians obeying the relation

$$\hat{H}_2=-\mathcal{M}\hat{H}_1^{\star}\mathcal{M}^{\dagger}, \tag{S10}$$

As the spectra of two subsystems are identical up to a minus sign, the composite system has a large zero-energy subspace, spanned by pairs of eigenstates with energies E and $-E$. Provided that $\mathcal{M}$ maps each site in subsystem 1 to a single site in subsystem 2, as in Eq. (S10) (i.e., with no global term mixing all sites), then the zero-energy subspace contains a rainbow state,

$$|\mathbf{I}\rangle=\frac{1}{\sqrt{\mathcal{D}_1}}\bigotimes_{j=1}^{M}\sum_{\sigma_j}|\sigma_j\rangle\otimes(\mathcal{M}\,|\sigma_j\rangle), \tag{S11}$$

where $\mathcal{D}_1$ denotes the number of states in the subsystem 1 and σ_j denotes the state of site j in it. The state $|\mathbf{I}\rangle$ is simply a tensor product of Bell pairs, and it has maximal entanglement between the two subsystems. Importantly, $|\mathbf{I}\rangle$ is an eigenstate of the full system, *independently* of the microscopic details of $\hat{H}_1$ (*33*). One can then make the two subsystems interact by adding a term $\hat{H}_{\text{int}}$. If it is chosen such that it has $|\mathbf{I}\rangle$ as an eigenstate, $|\mathbf{I}\rangle$ will be a scarred eigenstate of the full system while other states generically become ergodic.

In our case, subsystem 1 is the top row of the ladder while subsystem 2 is the bottom row. The transformation related to their Hamiltonians is

$$\mathcal{M}=\left.\prod_{k=1}^{M}\hat{\mathbf{d}}_k^{x}\right)\hat{\mathcal{P}}_{\mathbf{d}\leftrightarrow\mathbf{u}}, \tag{S12}$$

where $\hat{\mathcal{P}}_{\mathbf{d}\leftrightarrow\mathbf{u}}$ is the operator exchanging the top and bottom row and the term between parentheses is a particle-hole exchange. The rainbow state can then be written succinctly as

$$|\mathbf{I}\rangle \equiv |E_M\rangle = \bigotimes_{j=1}^{M} \frac{1}{\sqrt{2}} \left(|{}^{\bullet}_{\circ}\rangle + |{}^{\circ}_{\bullet}\rangle \right) \equiv |\mathbf{TTT}\ldots\mathbf{TT}\rangle \,, \tag{S13}$$

which reveals the structure of Bell pairs formed between the two rungs of the ladder. Upon addition of $\hat{H}_{\text{int}}$, the rainbow state $|I\rangle$ remains an exact eigenstate with energy MJ_a, thus becoming a rainbow scar of the full model.

The previous construction is not limited to a single state. We can generate additional rainbow scar states by acting on $|\mathbf{I}\rangle$ with an operator $\hat{O} \otimes \mathbb{1}_2$, where $\hat{O}$ commutes with the Hamiltonian $\hat{H}_1$ (*33*). The resulting state, by construction, still belongs to the zero-energy subspace of the combination of $\hat{H}_1$ and $\hat{H}_2$. Provided that the resulting state is also an eigenstate of $\hat{H}_{\text{int}}$, it is then a scarred eigenstate of the full system. Two different families of scars in our model in Eq. (1) can be built this way by choosing different operators $\hat{O}$. Moreover, it allows to get simple product states with overlap only on these special eigenstates.

5.1 First scar family

To construct the first scar family, we use the operator $\hat{Z} = \sum_{k=1}^{M} \hat{\mathbf{u}}_k^z$. As explained in the main text, this operator commutes with $\hat{H}_1$ and it can be applied up to M times, changing triplets into singlets and vice versa. The resulting states, $|E_n\rangle$, are members of the first type of scar family. They represent a symmetric superposition of all configurations with a fixed number of singlets and triplets, and it is straightforward to verify that they are eigenstates of the model with energy $E_n = J_a(2n - M)$.

The underlying structure of the first family of scarred states $|E_n\rangle$ is the restricted spectrum generating algebra (*28*). This is seen by noting that they can be built using the raising operator

$\hat{S}^+ = \sum_{k=1}^{M} |T_k\rangle \langle S_k|$. Thus, we can equivalently express the first type of scar states as

$$|E_n\rangle = \frac{1}{\mathcal{N}}(\hat{S}^+)^n |E_0\rangle = \frac{1}{\mathcal{N}}(\hat{S}^+)^n |\mathbf{SS}\ldots\mathbf{S}\rangle\,, \tag{S14}$$

where $\mathcal{N}$ is a normalization constant. We recognize that these scarred eigenstates are built in a similar way to previously known examples, e.g., in the spin-1 XY model (*27*). In Sec. 9 we compute their entanglement entropy for the bipartition perpendicular to the ladder, and we find that the eigenstate at zero energy has $S_{1,\perp} = 0.5 + 0.5\log(\pi M/8)$, in the limit $M \to \infty$. We note that the su(2) algebraic structure of these states implies they have extensive multipartite entanglement (*13, 14*).

5.2 Second scar family

The second scar family can be built recursively from the first family, where the role of the operator $\hat{O}$ in the rainbow construction is played by the subsystem Hamiltonian $\hat{H}_1$. Specifically, the second type of scar states are given by raising from $|E_{n-1}\rangle$ according to

$$|E_n'\rangle \propto \hat{P}^Q_{M-2n}\left[\hat{H}_1 - \left(\sum_k \frac{\omega_k}{M}\right)\hat{Z}\right]|E_{n-1}\rangle\,, \tag{S15}$$

or, equivalently, by lowering from $|E_{n+1}\rangle$ as

$$|E_n'\rangle \propto \hat{P}^Q_{M-2n}\left[\hat{H}_1 - \left.\sum_k \frac{\omega_k}{M}\right)\hat{Z}\right]|E_{n+1}\rangle\,. \tag{S16}$$

In these expressions, $\hat{P}^Q_q$ is a projector on the sector of $\hat{Q}$ with eigenvalue q.

In order to write the states $|E_n'\rangle$ in a more explicit form, let us define ensembles of sites $\Lambda = \{1, 2, \ldots M\}$, $\Lambda_k = \{k, k+1\}$ and $\Lambda_{\overline{k}} = \Lambda \setminus \Lambda_k$. With this, we can express $|E_n'\rangle$ as

$$\begin{aligned}|E_n'\rangle =& \sum_{k=1}^{M-1} \frac{J_{e,k}}{2\mathcal{N}_n}\left(|\mathbf{HD}\rangle_{\Lambda_k} + |\mathbf{DH}\rangle_{\Lambda_k}\right) \otimes |M-n-1, n-1\rangle_{\Lambda_{\overline{k}}} \\ &+ \frac{1}{\mathcal{N}_n}\left(\sum_{k=1}^{M} \hat{T}_k \overline{\omega}_k\right)|M-n, n\rangle_{\Lambda}\,,\end{aligned} \tag{S17}$$

with $n = 1, 2, \dots, M-1$, $\overline{\omega}_j = \omega_j - \frac{1}{M}\sum_{k=1}^{M}\omega_k$, and $|a,b\rangle_A$ the symmetric superposition of all states with a singlets and b triplets on sites in the ensemble A. More details about the construction of these states and the proof that they are eigenstates of the model are given in Secs. 6-7, where we also derive the normalization factors $\mathcal{N}_n$. There, we prove that $|E'_n\rangle$ have the same energies as the first family of scar states, i.e., $E'_n = J_a(2n - M)$.

To the best of our knowledge, there is no su(2) algebraic construction for the second family of scarred states $|E'_n\rangle$, despite their equal energy spacing. Indeed, the latter is generated by acting with an operator on $|E_n\rangle$, and not on $|E'_{n-1}\rangle$ or $|E'_{n+1}\rangle$. Moreover, the second type of scarred eigenstates depend sensitively on the parameters $J_{e,k}$ and ω_k of the model. Therefore, the entanglement of these states is not fixed, like in the first family of scars, as we discuss in detail in Sec. 9. For a cut perpendicular to the ladder, the second type of scarred states generally possess entanglement entropy that scales logarithmically with subsystem size. While we are unable to rule out the possibility of volume-law entanglement scaling, our analytical and numerical results strongly suggest that, in the large M limit, the second type of scarred state with zero energy obeys $S_{\perp,1} < S_{\perp,2} < S_{\perp,1} + \log 4$ (*35*). This suggests it should always be possible to find a low-entangled state that overlap only on the scarred stats of the second family, regardless of the values of parameters. By tuning the hopping $J_{e,k}$ one can then change which state has this anomalous overlap, as we will demonstrate in Sec. 8.

5.3 Conditions on the Hamiltonian parameters

With some insights into the structure of both families of scarred eigenstates, we can now check if some conditions on the Hamiltonian parameters could potentially be relaxed. As the only parameters with no disorder are the J_a, it is natural to ask if we are allowed to introduce inhomogeneity in these couplings while preserving the constructed scarred states. Randomizing J_a can be easily shown not to work. Recall from the rainbow scar construction, the scarred state

$|E_n\rangle$ of the first family is simply the symmetric superposition of all combinations with $M - n$ singlets and n triplets. In order for these states to be eigenstates of the full system, they also need to be eigenstates of $\hat{H}_{\text{int}}$, which encompasses the action of the vertical XY terms. The states $|\mathbf{SSSS}\ldots\rangle$ and $|\mathbf{TTTT}\ldots\rangle$ are eigenstates even if the J_a are disordered, but their energy will change. However, the other eigenstates will generally *not* be eigenstates of $\hat{H}_{\text{int}}$ unless the J_a are equal.

Indeed, let us take the state with 3 dimers, $\frac{1}{2}\left(|\mathbf{STT}\rangle + |\mathbf{TST}\rangle + |\mathbf{TTS}\rangle\right)$, as an example. Applying $\hat{H}_{\text{int}}$ to it leads to a different prefactor for each term in the superposition. The first term will have a prefactor of $-J_{a,1} + J_{a,2} + J_{a,3}$, the second term a prefactor of $J_{a,1} - J_{a,2} + J_{a,3}$, and so on. This state is therefore not an eigenstate of $\hat{H}_{\text{int}}$ unless all the J_a are equal. Importantly, it is also not possible to create eigenstates of $\hat{H}_{\text{int}}$ by linear combinations of the $|E_n\rangle$. The same reasoning holds for the second family of scarred states. In the end, if the J_a are disordered, the model only has two exact scarred eigenstates, $|E_0\rangle$ and $|E_N\rangle$, which are incidentally the only states in the sectors $Q = -N/2$ and $Q = N/2$.

Other ways of relaxing the constraints on the Hamiltonian parameters are less obvious as the $J_{e,k}$ and ω_k are already disordered. Since the rainbow scars construction rests on the similarity between the top and bottom chains, it is not possible to use different disorder realizations for each. However, one might ask if perhaps a different mirror transformation $\hat{\mathcal{M}}$ relating the two rows could lead to a simpler Hamiltonian. In particular, as it is difficult to engineer a superconducting chip capable of both strong positive and negative couplings, it would be desirable to have positive J_e coupling on both chains. In that case, these two parts of the Hamiltonian can satisfy the rainbow scar construction by using

$$\mathcal{M} = \left(\hat{\mathbf{d}}_1^x \hat{\mathbf{d}}_2^y \hat{\mathbf{d}}_3^x \hat{\mathbf{d}}_4^y \hat{\mathbf{d}}_5^x \ldots \hat{\mathbf{d}}_M^y\right) \hat{\mathcal{P}}_{\mathbf{d}\leftrightarrow\mathbf{u}}, \tag{S18}$$

and the system governed by $\hat{H}_{\mathbf{u}} + \hat{H}_{\mathbf{d}}$ will also have rainbow scars in that case. However, the

key difference is the structure of the scarred eigenstates and what happens when they are acted upon by the vertical XY terms of $\hat{H}_{\text{int}}$.

Due to the nature of the mirror transformation in Eq. (S18), the rainbow state will now be equal to $|\mathbf{TSTS}\ldots\rangle$, up to an irrelevant overall phase. We can then create the first family of scars by acting on this state with the total magnetization on the top chain $\hat{Z}$ that exchanges triplets and singlets, as these states will also be zero-energy eigenstates of $\hat{H}_{\mathbf{u}} + \hat{H}_{\mathbf{d}}$. Starting from the state $|\mathbf{TSTS}\ldots\rangle$ and generating a basis of this subspace using a Lanczos procedure, we find the following states in a simple example with $M = 3$:

$$
\begin{aligned}
|S_0\rangle &= |\mathbf{TST}\rangle \\
|S_1\rangle &= \frac{1}{\sqrt{3}}\left(|\mathbf{SST}\rangle + |\mathbf{TTT}\rangle + |\mathbf{TSS}\rangle\right) \\
|S_2\rangle &= \frac{1}{\sqrt{3}}\left(|\mathbf{TTS}\rangle + |\mathbf{SSS}\rangle + |\mathbf{STT}\rangle\right) \\
|S_3\rangle &= |\mathbf{STS}\rangle\,.
\end{aligned}
\tag{S19}
$$

These states will be eigenstates of the full system only if they are eigenstates of $\hat{H}_{\text{int}}$. A quick check shows that this is only the case for $|S_0\rangle$ and $|S_3\rangle$. For all other states, the terms in the superposition have a different number of triplets and singlets, leading to a different prefactor. As the states are also non-overlapping, it is not possible to create eigenstates of $\hat{H}_{\text{int}}$ by linear combinations of the $|S_n\rangle$. Thus, in the full system, only $|S_0\rangle$ and $|S_M\rangle$ survive as exact eigenstates. In fact, these exact states were previously found in Ref. (*46*) which studied the model with lower and upper J_e equal. One way to reinstate the $|S_n\rangle$ as eigenstates would be by staggering the J_a between odd and even bonds. However, this does not solve the problem of having large positive and negative couplings. The resulting model would also be equivalent to the one we study, up to a rotation around the Z axis on every other spin of the lower chain.

In summary, there are crucial conditions on the couplings on the model that are due to the important role of $\hat{H}_{\text{int}}$ that connects the two copies of the system. While the prerequisite of the rainbow scar construction – an opposite spectrum between two halves of a system – is met in

many systems, the additional condition that these eigenstates be preserved by $\hat{H}_{\text{int}}$ is usually what precludes the existence of this type of scars. Additionally, acting on these states with $\hat{H}_{\mathbf{u}}$ will always lead to other zero-energy eigenstates of $\hat{H}_{\mathbf{u}} + \hat{H}_{\mathbf{d}}$, but it is even harder to ensure that such states would be eigenstates of $\hat{H}_{\text{int}}$ (especially in the presence of disorder). To the best of our knowledge, our model is the first where this has been shown to occur.

6 Building scarred states of the second family

In this section, we derive the exact form of the scarred states of the second family. The formal proof that these states are eigenstates of the model is delegated to the subsequent section.

In order to reveal the structure of the second family of scarred states, let us first derive the action of $\hat{H}_1 - \left(\sum_k \omega_k / M\right) \hat{Z}$ in the dimer basis. First, we define

$$\hat{h}^{\|,1}_{k,k+1} = \frac{J_{e,k}}{2} \left(\hat{\mathbf{u}}^x_k \hat{\mathbf{u}}^x_{k+1} + \hat{\mathbf{u}}^y_k \hat{\mathbf{u}}^y_{k+1} \right). \tag{S20}$$

From there it is straightforward to see that

$$\hat{H}_1 - \quad \sum_k \frac{\omega_k}{M} \Bigg) \hat{Z} = \sum_{k=1}^{M-1} \hat{h}^{\|,1}_{k,k+1} + \sum_{k=1}^{M} \overline{\omega}_k \mathbf{u}^z_k, \tag{S21}$$

where $\overline{\omega}_k = \omega_k - \left(\sum_i \omega_i / M\right)$. As we will only apply this operator to scarred states of the first family, which contain no $|\mathbf{D}\rangle$ or $|\mathbf{H}\rangle$, we can ignore any configurations containing them. The action of $\hat{h}^{\|,1}_{k,k+1}$ and $\mathbf{u}^z_k$ on dimers is then

$$\begin{aligned}
\hat{h}^{\|,1}_{k,k+1} |\mathbf{TT}\rangle &= \frac{1}{2} J_e (|\mathbf{HD}\rangle + |\mathbf{DH}\rangle), \\
\hat{h}^{\|,1}_{k,k+1} |\mathbf{SS}\rangle &= -\frac{1}{2} J_e (|\mathbf{HD}\rangle + |\mathbf{DH}\rangle), \\
\hat{h}^{\|,1}_{k,k+1} |\mathbf{TS}\rangle &= \frac{1}{2} J_e (|\mathbf{HD}\rangle - |\mathbf{DH}\rangle), \\
\hat{h}^{\|,1}_{k,k+1} |\mathbf{ST}\rangle &= -\frac{1}{2} J_e (|\mathbf{HD}\rangle - |\mathbf{DH}\rangle), \\
\overline{\omega}_k \hat{\mathbf{u}}^z_k |\mathbf{S}\rangle &= -\overline{\omega}_k |\mathbf{T}\rangle, \\
\overline{\omega}_k \hat{\mathbf{u}}^z_k |\mathbf{T}\rangle &= -\overline{\omega}_k |\mathbf{S}\rangle.
\end{aligned} \tag{S22}$$

From there, we immediately see that

$$\hat{h}^{\|,1}_{k,k+1}(|\mathbf{TS}\rangle + |\mathbf{ST}\rangle) = 0. \tag{S23}$$

To represent the symmetric superposition, we recall the notation introduced to describe scarred states of the first family:

$$|E_n\rangle = \frac{1}{\mathcal{N}}\,|L{-}n, n\rangle = \frac{1}{\mathcal{N}} \sum_{|\phi\rangle\in(L-n,n)} |\phi\rangle\,. \tag{S24}$$

This state is a fully symmetric superposition of all configurations on L sites with $L-n$ singlets and n triplets. For brevity, we will first work out what happens when we apply $\hat{h}^{\|,1}_{1,2}$ (the same is true for any $\hat{h}^{\|,1}_{k,k+1}$):

$$\begin{aligned}
\hat{h}^{\|,1}_{1,2}\,|E_{n-1}\rangle =& \frac{\hat{h}^{\|,1}_{1,2}}{\mathcal{N}}\,|M{-}n{+}1, n{-}1\rangle \\
=& \frac{\hat{h}^{\|,1}_{1,2}}{\mathcal{N}}\Big[(|\mathbf{TS}\rangle + |\mathbf{ST}\rangle)\,|M{-}n, n{-}2\rangle \\
&+ |\mathbf{TT}\rangle\,|M{-}n{+}1, n{-}3\rangle \\
&+ |\mathbf{SS}\rangle\,|M{-}n{-}1, n{-}1\rangle\Big] \\
=& \frac{J_{e,1}}{2\mathcal{N}}(|\mathbf{HD}\rangle + |\mathbf{DH}\rangle)\,|M{-}n{+}1, n{-}3\rangle \\
&- \frac{J_{e,1}}{2\mathcal{N}}(|\mathbf{HD}\rangle + |\mathbf{DH}\rangle)\,|M{-}n{-}1, n{-}1\rangle\,,
\end{aligned} \tag{S25}$$

where we used the condition (S23) to cancel the contribution of the first term. Applying the projector $\hat{P}^{Q}_{M-2n}$ singles out one of the terms:

$$\hat{P}^{Q}_{M-2n}\hat{h}^{\|,1}_{1,2}\,|E_{n-1}\rangle = -\frac{J_{e,1}}{2\mathcal{N}}(|\mathbf{HD}\rangle + |\mathbf{DH}\rangle)\,|M{-}n{-}1, n{-}1\rangle\,. \tag{S26}$$

Next, we look at the action of $\overline{\omega}_1\hat{\mathbf{u}}^z_1$:

$$\begin{aligned}
\overline{\omega}_1\hat{\mathbf{u}}^z_1\,|E_{n-1}\rangle =& \frac{\overline{\omega}_1\hat{\mathbf{u}}^z_1}{\mathcal{N}}\,|M{-}n{+}1, n{-}1\rangle \\
=& \frac{\overline{\omega}_1\hat{\mathbf{u}}^z_1}{\mathcal{N}}\Big[\,|\mathbf{T}\rangle\,|M{-}n{+}1, n{-}2\rangle + |\mathbf{S}\rangle\,|M{-}n, n{-}1\rangle\Big] \\
=& \frac{-\overline{\omega}_1}{\mathcal{N}}\Big[\,|\mathbf{S}\rangle\,|M{-}n{+}1, n{-}2\rangle + |\mathbf{T}\rangle\,|M{-}n, n{-}1\rangle\Big].
\end{aligned} \tag{S27}$$

Applying the projector in this case gives

$$\hat{P}^{Q}_{M-2n}\overline{\omega}_1\hat{\mathbf{u}}^z_1\left|E_{n-1}\right\rangle = -\,\frac{\overline{\omega}_1}{\mathcal{N}}\left|\mathbf{T}\right\rangle\left|M{-}n,n{-}1\right\rangle. \tag{S28}$$

The result is similar if we act on another site k, with a prefactor $\overline{\omega}_k$ and a triplet on that site. Ultimately, we end up with a collection of all states with n singlets and $n-1$ triplets, but each of them has a prefactor that depends on the location of the triplets. Let us introduce the operator $\hat{T}_k$ that gives 1 if this site is a triplet and 0 otherwise. We can then write

$$\hat{P}^{Q}_{M-2n}\sum_{k=1}^{M}\overline{\omega}_k\hat{\mathbf{u}}^z_k\left|E_{n-1}\right\rangle = -\,\frac{1}{\mathcal{N}}\left.\sum_{k=1}^{M}\hat{T}_k\overline{\omega}_k\right)\left|M{-}n,n\right\rangle. \tag{S29}$$

To write down the scarred states of the second family, we now simply need to gather the terms from Eqs. (S26) and (S29). We also remove the overall minus sign and normalize the state. Let us introduce the ensembles of sites $\Lambda = \{1,2,\dots M\}$, $\Lambda_k = \{k,k+1\}$ and $\Lambda_{\overline{k}} = \Lambda - \Lambda_k = \{1,2\dots k-1,k+2,k+3,\dots M\}$. They represent, respectively, all sites, sites k and $k+1$, and all sites except k and $k+1$. This allows us to write $|E'_n\rangle$ as

$$\begin{aligned}|E'_n\rangle = &\sum_{k=1}^{M-1}\frac{J_{e,k}}{2\mathcal{N}_n}(|\mathbf{HD}\rangle_{\Lambda_k} + |\mathbf{DH}\rangle_{\Lambda_k})\otimes|M{-}n{-}1,n{-}1\rangle_{\Lambda_{\overline{k}}}\\ &+\frac{1}{\mathcal{N}_n}\left(\sum_{k=1}^{M}\hat{T}_k\overline{\omega}_k\right)|M-n,n\rangle_{\Lambda}\,,\end{aligned} \tag{S30}$$

with $n = 1,2,\dots,M-1$. As an example, let us write out the case for $M=4$ and $n=1$:

$$\begin{aligned}|E'_1\rangle = \frac{1}{2\mathcal{N}_1}\Big[&J_{e,1}(|\mathbf{DHSS}\rangle + |\mathbf{HDSS}\rangle) + J_{e,2}(|\mathbf{SDHS}\rangle\\ &+|\mathbf{SHDS}\rangle) + J_{e,3}(|\mathbf{SSDH}\rangle + |\mathbf{SSHD}\rangle)\Big]\\ +\frac{1}{\mathcal{N}_1}\Big[&\overline{\omega}_1\,|\mathbf{TSSS}\rangle + \overline{\omega}_2\,|\mathbf{STSS}\rangle\\ &+\overline{\omega}_3\,|\mathbf{SSTS}\rangle + \overline{\omega}_4\,|\mathbf{SSST}\rangle\Big].\end{aligned} \tag{S31}$$

Finally, let us show that the same result is obtained if we generate $|E'_n\rangle$ by lowering from $|E_{n+1}\rangle$. We have

$$\hat{P}^{Q}_{M-2n}\hat{h}^{\|,1}_{1,2}\left|E_{n+1}\right\rangle = \frac{J_{e,1}}{2\mathcal{N}}(|\mathbf{HD}\rangle + |\mathbf{DH}\rangle)\left|M{-}n{-}1,n{-}1\right\rangle. \tag{S32}$$

and

$$\hat{P}^{Q}_{M-2n}\overline{\omega}_1\hat{\mathbf{u}}^z_1 \left|E_{n+1}\right\rangle = \frac{-\overline{\omega}_1}{\mathcal{N}} \left|\mathbf{S}\right\rangle \left|M-n-1, n\right\rangle . \tag{S33}$$

From the latter equation, we can derive that

$$\hat{P}^{Q}_{M-2n}\sum_{k=1}^{M}\overline{\omega}_k\hat{\mathbf{u}}^z_k \left|E_{n-1}\right\rangle = \frac{-1}{\mathcal{N}} \left.\sum_{k=1}^{M}\hat{S}_k\overline{\omega}_k\right) \left|M-n, n\right\rangle , \tag{S34}$$

where $\hat{S}_k$ gives 1 if this site is a triplet and 0 otherwise. Now we can notice that $\left|M-n, n\right\rangle$ is composed entirely of triplets and singlets. Consequently, $(\hat{T}_k + \hat{S}_k) = \mathbf{1}_k$ when acting on that state. Moreover, we know that the $\overline{\omega}$ must sum to 0 by construction. This allows us to state that

$$\sum_{k=1}^{M}\overline{\omega}_k(\hat{S}_k+\hat{T}_k)\left|M-n, n\right\rangle = \sum_{k=1}^{M}\overline{\omega}_k\left|M-n, n\right\rangle = 0. \tag{S35}$$

From there we can conclude that

$$\begin{aligned}\hat{P}^{Q}_{M-2n}\sum_{k=1}^{M}\overline{\omega}_k\hat{\mathbf{u}}^z_k \left|E_{n-1}\right\rangle &= \frac{-1}{\mathcal{N}} \left.\sum_{k=1}^{M}\hat{S}_k\overline{\omega}_k\right) \left|M-n, n\right\rangle \\ &= \frac{1}{\mathcal{N}} \left.\sum_{k=1}^{M}\hat{T}_k\overline{\omega}_k\right) \left|M-n, n\right\rangle .\end{aligned} \tag{S36}$$

Gathering the results of Eqs. (S32) and (S36) we find the same result as in Eq. (S30).

6.1 Normalization

As we have the exact wavefunction for the scarred states of the second family, we can compute their normalization factor $\mathcal{N}_n$. It admits a simple expression

$$\mathcal{N}_n = \sqrt{\binom{M-2}{n-1}\left[\frac{1}{2}\sum_{k=1}^{M-1}J^2_{e,k} + \sum_{k=1}^{M}\overline{\omega}^2_k\right]}. \tag{S37}$$

This expression does not contain any cross-term $\overline{\omega}_k\overline{\omega}_j$ because all possible combinations of $k \neq j$ appear and we can then express them as

$$\sum_{k=1}^{M-1}\sum_{j=k+1}^{M}\overline{\omega}_k\overline{\omega}_j = -\frac{1}{2}\sum_{k=1}^{M}\overline{\omega}^2_k, \tag{S38}$$

by using the fact that $\sum_{k=1}^{M}\overline{\omega}_k = 0$ and as such

$$0 = \quad \sum_{k=1}^{M}\overline{\omega}_k\Bigg)^2 = \sum_{k=1}^{M}\overline{\omega}_k^2 + 2\sum_{k=1}^{M-1}\sum_{j=k+1}^{M}\overline{\omega}_k\overline{\omega}_j. \tag{S39}$$

7 Proof that scarred states are eigenstates

In this section, we prove that the two families of scarred states, written down in the main text, are eigenstates of the model in Eq. (S4). We first address the straightforward cases of the first family of scars with $n = 0$ and $n = M$. We then show the proof for the slightly more complicated $n = 1$ case and finally demonstrate that the same arguments generalize to arbitrary n.

7.1 $n = 0$ and $n = M$ scarred states

Proving that $|E_0\rangle = |\mathbf{S}\ldots\mathbf{S}\rangle$ is an eigenstate is trivial, as we know that $|\mathbf{SS}\rangle$ is an eigenstate of $\hat{h}^{\parallel}_{k,k+1}$ with energy 0 and $|\mathbf{S}\rangle$ is an eigenstates of $\hat{h}^{\perp}_{k}$ with energy $-J_a$. Thus, $|E_0\rangle$ must be an eigenstate of $\hat{H}$ with energy $-MJ_a$. Similarly, $|\mathbf{TT}\rangle$ is an eigenstate of $\hat{h}^{\parallel}_{k,k+1}$ with energy 0 and $|\mathbf{T}\rangle$ is an eigenstate of $\hat{h}^{\perp}_{k}$ with energy J_a. Thus, $|E_M\rangle = |\mathbf{TT}\ldots\mathbf{T}\rangle$ must be an eigenstate of $\hat{H}$ with energy MJ_a.

7.2 $n = 1$ scarred states

For $n = 1$ we will prove the eigenstate property by considering a 2×3 ladder and then show that the same holds for larger systems. Consider the state

$$\begin{aligned} |\psi\rangle &= \beta_1\left(|\mathbf{HDS}\rangle + |\mathbf{DHS}\rangle\right) + \beta_2\left(|\mathbf{SHD}\rangle + |\mathbf{SDH}\rangle\right) \\ &\quad + \alpha_1|\mathbf{TSS}\rangle + \alpha_2|\mathbf{STS}\rangle + \alpha_3|\mathbf{SST}\rangle\,. \end{aligned} \tag{S40}$$

Applying the Hamiltonian to this state and after some algebra, we obtain

$$
\begin{aligned}
\hat{H}\left|\psi\right\rangle &= -J_a\left|\psi\right\rangle \\
&+ \left[2\beta_1\left(\omega_1 - \omega_2\right) + J_{e,1}\left(\alpha_2 - \alpha_1\right)\right]\left(\left|\mathbf{DHS}\right\rangle - \left|\mathbf{HDS}\right\rangle\right) \\
&+ \left[2\beta_2\left(\omega_2 - \omega_3\right) + J_{e,2}\left(\alpha_3 - \alpha_2\right)\right]\left(\left|\mathbf{SDH}\right\rangle - \left|\mathbf{SHD}\right\rangle\right) \\
&+ \left(J_{e,2}\beta_1 - J_{e,1}\beta_2\right)\left(\left|\mathbf{HTD}\right\rangle - \left|\mathbf{DTH}\right\rangle\right).
\end{aligned}
\tag{S41}
$$

For $\left|\psi\right\rangle$ to be an eigenstate, the coefficients must obey

$$2\beta_1\left(\omega_1 - \omega_2\right) + J_{e,1}\left(\alpha_2 - \alpha_1\right) = 0, \tag{S42}$$

$$2\beta_2\left(\omega_2 - \omega_3\right) + J_{e,2}\left(\alpha_3 - \alpha_2\right) = 0, \tag{S43}$$

$$J_{e,2}\beta_1 - J_{e,1}\beta_2 = 0. \tag{S44}$$

In general, we have 5 unknowns but only 3 equations, leaving room for two non-trivial solutions. The first option is to set all α_j to be equal and all β_j to zero:

$$\alpha_j = 1, \quad \beta_j = 0. \tag{S45}$$

This corresponds to the scarred state of the first family, $\left|E_1\right\rangle$. The other solution is given by the scarred states of the second family that obey (up to a normalization factor)

$$\alpha_j = \omega_j - \frac{1}{M}\sum_k \omega_k = \overline{\omega}_j, \quad \beta_j = \frac{J_{e,j}}{2}. \tag{S46}$$

Furthermore, we can verify that the two families of scars are orthogonal as their overlap is given by

$$\sum_j \overline{\omega}_j = \sum_j \left. \omega_j - \frac{1}{M}\sum_k \omega_k \right) = 0. \tag{S47}$$

This completes the proof for the special case of $M = 3$. However, generalizing this to an arbitrary M is now straightforward. The general state is

$$\begin{aligned}
|\psi\rangle &= \beta_1 \left(|\mathbf{HDS}\ldots\mathbf{S}\rangle + |\mathbf{DHS}\ldots\mathbf{S}\rangle\right) \\
&+ \beta_2 \left(|\mathbf{S}\ldots\mathbf{SHDS}\rangle + |\mathbf{S}\ldots\mathbf{SDHS}\rangle\right) \\
&+ \ldots + \beta_{M-2} \left(|\mathbf{S}\ldots\mathbf{SHD}\rangle + |\mathbf{S}\ldots\mathbf{SDH}\rangle\right) \\
&+ \beta_{M-1} \left(|\mathbf{HDS}\ldots\mathbf{S}\rangle + |\mathbf{DHS}\ldots\mathbf{S}\rangle\right) \\
&+ \alpha_1 |\mathbf{TS}\ldots\mathbf{S}\rangle + \alpha_2 |\mathbf{STS}\ldots\mathbf{S}\rangle + \ldots \\
&+ \alpha_{M-1} |\mathbf{S}\ldots\mathbf{STS}\rangle + \alpha_M |\mathbf{S}\ldots\mathbf{ST}\rangle .
\end{aligned} \tag{S48}$$

Now we have M different α_j and $M-1$ different β_j, so $2M-1$ unknowns in total. For each block of 2×2 sites we get an equation similar to Eqs. (S42) and (S43). Thus, for $j = 1$ to $M-1$ we have

$$2\beta_j \left(\omega_j - \omega_{j+1}\right) + J_{e,j} \left(\alpha_{j+1} - \alpha_j\right) = 0. \tag{S49}$$

For each rectangular block of 2×3 sites we get an equation similar to Eq. (S44). Hence, for $j = 1$ to $M-2$ we have

$$J_{e,j+1}\beta_j - J_{e,j}\beta_j + 1 = 0. \tag{S50}$$

For a chain with $2M$ sites, this yields $(M-1) + (M-2) = 2M-3$ equations. So for any system size we always get at least two scarred states with energy $-(M-2)J_a$. It is easy to check that the two solutions given in Eqs. (S45) and (S46) are still valid.

7.3 Other values of n

While we treated the case $n = 1$ on its own to provide a simple example, the recipe is exactly the same for general n (except for $n = 0$ and $n = M$ that were already proven). We use the same Ansatz in which we only consider states with one $\mathbf{DH} + \mathbf{HD}$ pair in a background of $n-1$ triplets and $M-n-1$ singlets and states with n triplets and $M-n$ singlets.

We first restrict our investigation to an arbitrary location $k,\ k+1$ for the hole-doublon pair.

$$\begin{aligned}|\psi\rangle &= \beta_j \left(|\ldots \mathbf{XHDY} \ldots\rangle + |\ldots \mathbf{XDHY} \ldots\rangle\right) \\ &+\alpha_{TS} |\ldots \mathbf{XTSY} \ldots\rangle + \alpha_{ST} |\ldots \mathbf{XSTY} \ldots\rangle ,\end{aligned} \tag{S51}$$

where $\mathbf{X}$ and $\mathbf{Y}$ denote either $\mathbf{S}$ or $\mathbf{T}$. For $\hat{h}^{\perp}$, the contribution on all other sites except k and $k+1$ is diagonal and equal to $J_a(2n-M)$. Therefore, to prove that states are eigenstates with this energy, we need to prove that the action of the rest of the Hamiltonian annihilates the state. For $\hat{h}^{\parallel}_{k,k+1}$ we only have to care about the action on sites $k-1$ to $k+2$. Indeed, the rest of the state is composed of triplets and singlets. For any other pair, if it is $\mathbf{SS}$ or $\mathbf{TT}$, then it is annihilated by the action of the Hamiltonian. If, instead, it is $\mathbf{TS}$, then there exists another state in the superposition, with the same weight, that has $\mathbf{ST}$ instead. Therefore, their superposition is also annihilated by the Hamiltonian.

First, we can look at what happens if we act on the middle pair. This leads to

$$\begin{aligned}&2\beta_j \left(\omega_j - \omega_{j+1}\right) \left(|\ldots \mathbf{XDHY} \ldots\rangle - |\ldots \mathbf{XHDY} \ldots\rangle\right) \\ &+J_{e,j} \left(\alpha_{ST} - \alpha_{TS}\right) \left(|\ldots \mathbf{XDHY} \ldots\rangle - |\ldots \mathbf{XHDY} \ldots\rangle\right) ,\end{aligned} \tag{S52}$$

and so

$$2\beta_j \left(\omega_j - \omega_{j+1}\right) + J_{e,j} \left(\alpha_{ST} - \alpha_{TS}\right) = 0. \tag{S53}$$

We get a unique equation for every of the $M-1$ pair of sites and for every of the $\binom{M-2}{n-1}$ possible background configurations, where $n = 1$ to $M-1$ is the index of the scarred states.

Now we still need to look at the effect of $\hat{h}^{\parallel}$ on $\mathbf{XD}$, $\mathbf{XH}$ (as well as $\mathbf{HY}$ and $\mathbf{DY}$). For that, we need to also consider the $\mathbf{DH}$ and $\mathbf{HD}$ pair placed one site to the left:

$$\begin{aligned}&\beta_{j-1} \left(|\ldots \mathbf{HDXY} \ldots\rangle + |\ldots \mathbf{DHXY} \ldots\rangle\right) \\ &+\beta_j \left(|\ldots \mathbf{XHDY} \ldots\rangle + |\ldots \mathbf{XDHY} \ldots\rangle\right) .\end{aligned} \tag{S54}$$

Applying the Hamiltonian to these states leads to

$$\begin{aligned}&\beta_{j-1} J_{e,j} \left(|\ldots H\overline{\mathbf{X}}\mathbf{DY} \ldots\rangle - |\ldots D\overline{\mathbf{X}}\mathbf{HY} \ldots\rangle\right) \\ &-\beta_j J_{e,j-1} \left(|\ldots H\overline{\mathbf{X}}\mathbf{DY} \ldots\rangle + |\ldots D\overline{\mathbf{X}}\mathbf{HY} \ldots\rangle\right) ,\end{aligned} \tag{S55}$$

where $\overline{\mathbf{X}} = \mathbf{T}$ if $\mathbf{X} = \mathbf{S}$ and $\overline{\mathbf{X}} = \mathbf{S}$ is $\mathbf{X} = \mathbf{T}$. Hence we get an equation

$$\beta_{j-1} J_{e,j} - \beta_j J_{e,j-1} = 0, \tag{S56}$$

for $j = 1$ to $M - 2$. Thus, for the β coefficients, we always have $M - 1$ unknown and $M - 2$ equations. Furthermore, these equations take the form of Eq. (S56) and are *identical* for any value of n. Once again we recognize that setting all α equal and all β to 0 is a valid solution, and so the first family of scarred state is indeed an eigenstate. As for the $n = 1$, we also recognize that Eq. (S56) admits $\beta_j = J_{e,j}/2$ as a solution.

For the α, if we add a contribution of $\overline{\omega}_j$ for each site that has a triplet on site j as in the second family of scars, we recognise that α_{ST} and α_{TS} have the same contributions outside of site k and $k+1$. It is then easy to see that $\alpha_{ST} - \alpha_{TS} = \overline{\omega}_{j+1} - \overline{\omega}_j$ and that Eq. (S53) is satisfied, concluding our proof.

8 Dynamical signatures of two scar families

Both families of scarred states are evenly spaced in energy with spacing $2J_a$, hence they can detected by persistent revivals following the quench from a suitably chosen initial state. In this section, we identify such initial states for the two scar families and prove the existence of revivals by computing the overlap of initial states with scarred eigenstates derived in previous sections. We illustrate how these results can be used to enhance quantum revivals by modulating the coupling between the qubits.

8.1 Reviving initial state for the first scar family

To probe the first scar family, in the main text we used the state $|\Pi\rangle$ given by:

$$|\Pi\rangle = \left| \begin{smallmatrix} \bullet & \bullet & \cdots & \bullet \\ \circ & \circ & & \circ \end{smallmatrix} \right\rangle . \tag{S57}$$

This state must undergo perfect revivals due to the exact su(2) algebraic structure, as we now explain. From the raising operator $\hat{S}^{+} = \sum_k |\mathbf{T}_k\rangle \langle \mathbf{S}_k|$, we can infer $\hat{S}^{x} \propto \hat{S}^{+} + (\hat{S}^{+})^{\dagger}$ or, equivalently,

$$\hat{S}^{x} \;\propto\; \sum_k \Big(|\substack{\circ\\ \bullet}\rangle \langle \substack{\circ\\ \bullet}| - |\substack{\bullet\\ \circ}\rangle \langle \substack{\bullet\\ \circ}| \Big)_k . \tag{S58}$$

The corresponding $\hat{S}^{z}$ operator is given by $\hat{S}^{z} = \sum_k |\mathbf{T}_k\rangle \langle \mathbf{T}_k| - |\mathbf{S}_k\rangle \langle \mathbf{S}_k|$, which is easily seen to have the same action as $\hat{H}$ in this subspace. Hence, if we prepare the system in the lowest-weight state of $\hat{S}^{x}$, it will undergo perfect precession around an effective field in the z-direction. It is easy to see that our state $|\Pi\rangle$ is indeed the lowest weight state of Eq. (S58). The precession that starts out in $|\Pi\rangle$ state will result in perfect state transfer to the highest-weight state $|\Pi'\rangle = |\substack{\circ\,\circ\\ \bullet\,\bullet} \cdots \substack{\circ\\ \bullet}\rangle$.

The existence of revivals from the $|\Pi\rangle$ initial state can be shown more explicitly by computing the overlap $|\langle \Pi | E_n \rangle|^2$ with eigenstates of the first scar family. First, it will be important to observe that we obtain a simple product state when all possible combinations of singlets and triplets are summed up:

$$\sum_{n=0}^{M} |M-n, n\rangle = \bigotimes_{k=1}^{M} (|\mathbf{T}\rangle + |\mathbf{S}\rangle) = 2^{M/2} |\substack{\circ\\ \bullet} \cdots \substack{\circ\\ \bullet}\rangle , \tag{S59}$$

where in the second equality we made use of $|\mathbf{T}\rangle + |\mathbf{S}\rangle = \sqrt{2}\, |\substack{\circ\\ \bullet}\rangle$. Since $|E_n\rangle = \binom{M}{n}^{-1/2} |M-n, n\rangle$, it is easy to see that

$$|\Pi'\rangle = |\substack{\circ\\ \bullet} \cdots \substack{\circ\\ \bullet}\rangle = \sum_{n=0}^{M} \sqrt{\frac{\binom{M}{n}}{2^M}}\, |E_n\rangle . \tag{S60}$$

The same procedure can applied to the $|\Pi\rangle$ state by noting that $|\mathbf{T}\rangle - |\mathbf{S}\rangle = \sqrt{2}\, |\substack{\bullet\\ \circ}\rangle$. This means that there is now a factor of -1 for each singlet present, such that

$$|\Pi\rangle = |\substack{\bullet\\ \circ} \cdots \substack{\bullet\\ \circ}\rangle = \sum_{n=0}^{M} (-1)^{M-n} \sqrt{\frac{\binom{M}{n}}{2^M}}\, |E_n\rangle . \tag{S61}$$

Thus, the $|\Pi\rangle$ and $|\Pi'\rangle$ states only have overlap on the first family of scarred eigenstates. As the latter are regularly spaced in energy, the dynamics initialized in $|\Pi\rangle$ or $|\Pi'\rangle$ exhibits perfect revivals.

8.2 Reviving initial state for the second scar family

For scarred states of the second family, the identification of the reviving initial state $|\phi_L\rangle$ is more subtle as the eigenstates now depend on disorder realization and there is no apparent algebraic structure. However, we can once again appeal to the fact that the sum of all singlet and triplet configurations can be written as a simple state in the Fock basis. For any n, $|E_n'\rangle$ contain terms

$$\frac{J_{e,k}}{2\mathcal{N}_n}(|\mathbf{HD}\rangle_{\Lambda_k}+|\mathbf{DH}\rangle_{\Lambda_k})\otimes|M-n-1,n-1\rangle_{\Lambda_{\overline{k}}}, \tag{S62}$$

with $\mathcal{N}_n$ the normalization factor given in Eq. (S37). Recall that Λ_k denotes sites k and $k+1$ and $\Lambda_{\overline{k}}$ denotes all sites except k and $k+1$. As a consequence, summing all $|E_n'\rangle$ with a prefactor $\mathcal{N}_n$ gives

$$\begin{aligned}
&\sum_{n=1}^{M-1}\mathcal{N}_n\,|E_n'\rangle \\
&=\sum_{k=1}^{M}\frac{J_{e,k}}{2}(|\mathbf{HD}\rangle_{\Lambda_k}+|\mathbf{DH}\rangle_{\Lambda_k})\otimes\sum_{n=1}^{m-1}|M-n-1,n-1\rangle_{\Lambda_{\overline{k}}} \\
&+\left(\sum_{k=1}^{M}\overline{\omega}_k\hat{T}_k\right)\sum_{n=1}^{M-1}|M-n,n\rangle_{\Lambda} \\
&=\sum_{k=1}^{M}\frac{J_{e,k}}{2}(|\mathbf{HD}\rangle_{\Lambda_k}+|\mathbf{DH}\rangle_{\Lambda_k})\otimes 2^{\frac{M-2}{2}}\left|\begin{smallmatrix}\circ & \cdots & \circ\\ \bullet & & \bullet\end{smallmatrix}\right\rangle_{\Lambda_{\overline{k}}} \\
&+\left(\sum_{k=1}^{M}\overline{\omega}_k\hat{T}_k\right)\left(2^{M/2}\,|\Pi'\rangle-|T\cdots T\rangle-|S\cdots SS\rangle\right) \\
&=2^{\frac{M-4}{2}}\sum_{k=1}^{M}J_{e,k}(|\mathbf{HD}\rangle_{\Lambda_k}+|\mathbf{DH}\rangle_{\Lambda_k})\otimes\left|\begin{smallmatrix}\circ & \cdots & \circ\\ \bullet & & \bullet\end{smallmatrix}\right\rangle_{\Lambda_{\overline{k}}} \\
&+2^{M/2}\left(\sum_{k=1}^{M}\overline{\omega}_k\hat{T}_k\right)|\Pi'\rangle\,.
\end{aligned} \tag{S63}$$

In order to simplify this state and to make the orthogonality with $|E_n\rangle$ obvious, we will consider the initial state

$$
\begin{aligned}
|\phi'_J\rangle =&\frac{1}{\mathcal{Z}}\sum_{k=1}^{M} J_{e,k}(|\mathbf{HD}\rangle_{\Lambda_k}+|\mathbf{DH}\rangle_{\Lambda_k})\otimes\left|\begin{smallmatrix}\bullet & \cdots & \bullet\\ \circ & & \circ\end{smallmatrix}\right\rangle_{\Lambda_{\overline{k}}}\\
=&\frac{1}{\mathcal{Z}}\Bigg(J_{e,1}\left|\begin{smallmatrix}\bullet & \circ & \bullet & \cdots & \bullet\\ \bullet & \circ & \circ & & \circ\end{smallmatrix}\right\rangle + J_{e,1}\left|\begin{smallmatrix}\circ & \bullet & \bullet & \cdots & \bullet\\ \circ & \bullet & \circ & & \circ\end{smallmatrix}\right\rangle\\
&+ J_{e,2}\left|\begin{smallmatrix}\bullet & \bullet & \circ & \bullet & \cdots & \bullet\\ \circ & \bullet & \circ & \circ & & \circ\end{smallmatrix}\right\rangle + J_{e_2}\left|\begin{smallmatrix}\bullet & \circ & \bullet & \bullet & \cdots & \bullet\\ \circ & \circ & \bullet & \circ & & \circ\end{smallmatrix}\right\rangle\\
&+ J_{e,M-1}\left|\begin{smallmatrix}\bullet & \cdots & \bullet & \bullet & \circ\\ \circ & & \circ & \bullet & \circ\end{smallmatrix}\right\rangle + J_{e,M-1}\left|\begin{smallmatrix}\bullet & \cdots & \bullet & \circ & \bullet\\ \circ & & \circ & \circ & \bullet\end{smallmatrix}\right\rangle\Bigg),
\end{aligned}
\tag{S64}
$$

with the normalization factor $\mathcal{Z} = \sqrt{2\sum_{k=1}^{M-1} J_{e,k}^2}$. As the $|\phi'_J\rangle$ state only has overlap with states containing one doublon and one hole, it has zero overlap with the scarred states of the first family that have neither of those. As such, any non-trivial dynamics after a quench from this state must come from scarred states of the second family. We can compute this overlap exactly:

$$
|\langle\phi'_J|E'_n\rangle|^2 = \frac{\binom{M-2}{n-1}}{2^{M-2}}\frac{\sum_{k=1}^{M-1} J_{e,k}^2}{\left(\sum_{k=1}^{M-1} J_{e,k}^2 + 2\sum_{k=1}^{M} \overline{\omega}_k^2\right)},
\tag{S65}
$$

which leads to

$$
\sum_{n=1}^{M-1} |\langle\phi'_J|E'_n\rangle|^2 = \frac{\sum_{k=1}^{M-1} J_{e,k}^2}{\left(\sum_{k=1}^{M-1} J_{e,k}^2 + 2\sum_{k=1}^{M} \overline{\omega}_k^2\right)}.
\tag{S66}
$$

It is important to notice here that it is not the ω_k that enter this equation but their counterparts $\overline{\omega}_k$, with the mean removed. So if we draw the $J_{e,k}$ and ω_k from the same distribution $[\Delta-\delta, \Delta+\delta]$, if $\Delta \gg \delta$ then we will have that $J_{e,k}^2 \gg \overline{\omega}_k^2$ as the former is at the scale of Δ^2 but the latter at the scale of δ^2. In that case, the state $|\phi'_J\rangle$ will have total overlap of order $\left(1+\frac{2M\delta^2}{(M-1)\Delta^2}\right)^{-1}$ on the QMBSs of the second family, making these states the only relevant ones.

Similarly, we can also define

$$
\begin{aligned}
|\phi_J\rangle =& \frac{1}{\mathcal{Z}} \sum_{k=1}^{M} J_{e,k}(|\mathbf{HD}\rangle_{\Lambda_k} + |\mathbf{DH}\rangle_{\Lambda_k}) \otimes \left|\begin{smallmatrix} \circ & \cdots & \circ \\ \bullet & & \bullet \end{smallmatrix}\right\rangle_{\Lambda_{\overline{k}}} \\
=& \frac{1}{\mathcal{Z}} \Bigg(J_{e,1} \left|\begin{smallmatrix} \bullet\circ\circ & \cdots & \circ \\ \bullet\circ\bullet & & \bullet \end{smallmatrix}\right\rangle + J_{e,1} \left|\begin{smallmatrix} \circ\bullet\circ & \cdots & \circ \\ \circ\bullet\bullet & & \bullet \end{smallmatrix}\right\rangle \\
&+ J_{e,2} \left|\begin{smallmatrix} \circ\bullet\circ\circ & \cdots & \circ \\ \bullet\bullet\circ\bullet & & \bullet \end{smallmatrix}\right\rangle + J_{e_2} \left|\begin{smallmatrix} \circ\circ\bullet\circ & \cdots & \circ \\ \bullet\circ\bullet\bullet & & \bullet \end{smallmatrix}\right\rangle \\
&+ J_{e,M-1} \left|\begin{smallmatrix} \circ & \cdots & \circ\bullet\circ \\ \bullet & & \bullet\bullet\circ \end{smallmatrix}\right\rangle + J_{e,M-1} \left|\begin{smallmatrix} \circ & \cdots & \circ\circ\bullet \\ \bullet & & \bullet\circ\bullet \end{smallmatrix}\right\rangle \Bigg).
\end{aligned}
\tag{S67}
$$

This state obeys the same $|\langle\phi_J|E'_n\rangle|^2$ as Eq. (S65), but with

$$
\langle\phi_J|E'_n\rangle = (-1)^{M-1-n}\langle\phi'_J|E'_n\rangle. \tag{S68}
$$

If the J_e only have a small amount of disorder, we can also look at the homogeneous state

$$
\begin{aligned}
|\phi\rangle = \frac{1}{\mathcal{N}} \Bigg(& \left|\begin{smallmatrix} \bullet\circ\circ & \cdots & \circ \\ \bullet\circ\bullet & & \bullet \end{smallmatrix}\right\rangle + \left|\begin{smallmatrix} \circ\bullet\circ & \cdots & \circ \\ \circ\bullet\bullet & & \bullet \end{smallmatrix}\right\rangle \\
&+ \left|\begin{smallmatrix} \circ\bullet\circ\circ & \cdots & \circ \\ \bullet\bullet\circ\bullet & & \bullet \end{smallmatrix}\right\rangle + \left|\begin{smallmatrix} \circ\circ\bullet\circ & \cdots & \circ \\ \bullet\circ\bullet\bullet & & \bullet \end{smallmatrix}\right\rangle \\
&+ \left|\begin{smallmatrix} \circ & \cdots & \circ\bullet\circ \\ \bullet & & \bullet\bullet\circ \end{smallmatrix}\right\rangle + \left|\begin{smallmatrix} \circ & \cdots & \circ\circ\bullet \\ \bullet & & \bullet\circ\bullet \end{smallmatrix}\right\rangle \Bigg).
\end{aligned}
\tag{S69}
$$

Due to its homogeneous weights, it requires less fine-tuning to prepare. It is well suited for the situation considered before, where $[\Delta - \delta, \Delta + \delta]$ with $\Delta \gg \delta$.

Finally, for experimental implementations, it is preferable to use an initial state that requires fewest gates to prepare. Because of this, in the main text we have considered the simpler cousin $|\phi_L\rangle$ of the state $|\phi\rangle$:

$$
|\phi_L\rangle = \frac{1}{\mathcal{N}} \Bigg(\left|\begin{smallmatrix} \bullet\circ\circ & \cdots & \circ \\ \bullet\circ\bullet & & \bullet \end{smallmatrix}\right\rangle + \left|\begin{smallmatrix} \circ\bullet\circ & \cdots & \circ \\ \circ\bullet\bullet & & \bullet \end{smallmatrix}\right\rangle \Bigg). \tag{S70}
$$

For this state, the overlap with the scarred states of the second family is

$$
|\langle\phi_L|E'_n\rangle|^2 = \frac{\binom{M-2}{n-1}}{2^{M-2}} \frac{J_{e,1}^2}{\left(\sum_{k=1}^{M-1} J_{e,k}^2 + 2\sum_{k=1}^{M} \varpi_k^2\right)}. \tag{S71}
$$

It is important to note that $|\phi'_J\rangle$, $|\phi_J\rangle$, $|\phi\rangle$ and $|\phi_L\rangle$ only have overlap with states with exactly one hole and one doublon. As a consequence, they are completely orthogonal to scarred states of the first family which only have singlets and triplets.

8.3 Tunability of revivals

To confirm our analysis above, we have numerically computed the overlap of states $|\Pi\rangle$, $|\phi_J\rangle$ and $|\phi_L\rangle$ with eigenstates of the model in Eq. (1) in Fig. S4. All the states have predominant support on scarred eigenstates, either of the first family ($|\Pi\rangle$ state) or the second family ($|\phi_J\rangle$ and $|\phi_L\rangle$ states). We emphasize that, as scarred states of the first family have no doublons or holons, they are *exactly* orthogonal to $|\phi_J\rangle$ and $|\phi_L\rangle$. Thus, persistent revivals from the latter states provide unambiguous evidence for the second type of QMBS. Indeed, Fig. S5**A** shows that the initial states $|\Pi\rangle$, $|\phi_J\rangle$ and $|\phi_L\rangle$ lead to revivals of the wave function and slow growth of entanglement entropy when compared to random Fock basis states – clear signatures of scarring. The rainbow nature of $|E_n\rangle$ and $|E'_n\rangle$ is also apparent in the dynamics, as the growth of entropy shows a stark difference between two different cuts, shown in Figs. S5**B**-**C**.

While the revival fidelity from the $|\phi_L\rangle$ initial state in Fig. S5 is not particularly high, we can leverage the tunability of the second family of scarred states to enhance it. Indeed, from Eq. (S71) it directly follows that the projection of $|\phi_L\rangle$ state on the set of scarred eigenstates $|E'_n\rangle$ is given by

$$\sum_{n=1}^{M-1} |\langle \phi_L | E'_n \rangle|^2 = \frac{J_{e,1}^2}{\sum_{k=1}^{M-1} J_{e,k}^2 + 2\sum_{k=1}^{M} \overline{\omega}_k^2}. \tag{S72}$$

Thus, in the limit of $J_{e,1} \to \infty$, we recover perfect revivals. To illustrate the effect of $J_{e,1}$ on the dynamics, in Fig. S6 we compute the fidelity dynamics as $J_{e,1}$ is modulated by an amount $\Delta_1 \in [0, 12]$, both in the ideal model and the model with experimental imperfections. As expected, for the ideal model the revivals are close to perfect for $\Delta_1 = 12$. Nevertheless, even after including the imperfections present in the device and realistic values of parameters, we still

see a substantial improvement in the first revival peak. We emphasize that the model remains chaotic for the chosen range of Δ_1 values.

9 Entanglement entropy of scarred eigenstates

Due to the su(2) algebra, the first family of scarred eigenstates have the structure of angular momentum eigenstates. As a result, they have entanglement entropy scaling as $\propto \log M$. In fact, it is straightforward to compute their entanglement entropy analytically. Let us assume that M is even and equal to $2R$. For simplicity, we will concentrate on the state with $n = R$ which has exactly zero energy, as it has the highest entanglement entropy among all scarred states. It is easy to decompose the state $|E_R\rangle$ as

$$\begin{aligned}|E_R\rangle =& \binom{2R}{R}^{-1/2} |R, R\rangle \\ =& \binom{2R}{R}^{-1/2} \sum_{k=0}^{R} |R-k, k\rangle \otimes |k, R-k\rangle \\ =& \sum_{k=0}^{R} \frac{\binom{R}{k}}{\sqrt{\binom{2R}{R}}} |\psi_{1,k}\rangle \otimes |\psi_{2,k}\rangle ,\end{aligned} \tag{S73}$$

where $|\psi_{1,k}\rangle$, $|\psi_{2,k}\rangle$ are the normalized versions of $|R-k, k\rangle$ and $|k, R-k\rangle$, respectively. From the last expression, we recognize the prefactors as the Schmidt coefficients. Therefore, the entanglement spectrum has $R+1 = M/2+1$ non-zero values with

$$p_k = \frac{\binom{R}{k}^2}{\binom{2R}{R}}, \tag{S74}$$

for $k = 0, 1, \ldots, R$. In the large-M limit, one can perform a saddle-point approximation to arrive at the result $S_{1,\perp} = 0.5 + 0.5\log(\pi M/8)$, demonstrating the logarithmic scaling with system size.

For the second family of scars, the computation is more arduous as the entanglement entropy depends on the disorder realization. Here we provide an exact computation for a few extremal

cases. While we do not have proof that these are the cases with maximum and minimum entanglement entropy, they match the results of our numerical optimizations. First, we can focus on the $|J_{e,k}| \gg |\overline{\omega}_k|$ case, in which we assume that the $\overline{\omega}_k$ are negligible. Let us also set all $J_{e,k} = 1$ equal to 1, except for the middle one which we set to $J_{e,R} = J_e$. We can then find the Schmidt decomposition of this state as

$$
\begin{aligned}
|E'_R\rangle =& \frac{J_e}{2\mathcal{N}_R}\sum_{k=0}^{R-1} |R{-}1{-}k, k\rangle\, |\mathbf{D}\rangle \otimes |\mathbf{H}\rangle\, |k, R{-}1{-}k\rangle \\
&+\frac{J_e}{2\mathcal{N}_R}\sum_{k=0}^{R-1} |R{-}1{-}k, k\rangle\, |\mathbf{H}\rangle \otimes |\mathbf{D}\rangle\, |k, R{-}1{-}k\rangle \\
&+\frac{1}{2\mathcal{N}_R}\sum_{k=0}^{R-2} |\tilde{\psi}_{1,k}\rangle \otimes |k{+}1, R{-}1{-}k\rangle \\
&+\frac{1}{2\mathcal{N}_R}\sum_{k=0}^{R-2} |k{+}1, R{-}1{-}k\rangle \otimes |\tilde{\psi}_{2,k}\rangle\,,
\end{aligned} \tag{S75}
$$

with

$$
|\tilde{\psi}_{1,k}\rangle = \sum_{j=1}^{R-1} \Big(|\mathbf{DH}\rangle_{\Lambda_j} + |\mathbf{HD}\rangle_{\Lambda_j} \Big) \otimes |R{-}2{-}k, k\rangle_{\overline{\Lambda}_j} \tag{S76}
$$

$$
|\tilde{\psi}_{2,k}\rangle = \sum_{j=R+1}^{M-1} \Big(|\mathbf{DH}\rangle_{\Lambda_j} + |\mathbf{HD}\rangle_{\Lambda_j} \Big) \otimes |R{-}2{-}k, k\rangle_{\overline{\Lambda}_j}\,, \tag{S77}
$$

where Λ_j denotes sites j and $j+1$ while $\overline{\Lambda}_j$ denote all other sites in the same half-system. To find the Schmidt coefficient the only step left is to normalize each ket in the decomposition. We already see that we have at most $4R - 2 = 2M - 2$ non-zero coefficients, showing that a state of this form can have, at most, entanglement growing as $\log M$.

Let us first write down $\mathcal{N}_R$ from Eq. (S37) as

$$
\mathcal{N}_R = \frac{1}{\sqrt{2}}\sqrt{\binom{2R-2}{R-1}\,(J_e^2 + M - 2)}. \tag{S78}
$$

Consequently, the contribution to the entanglement spectrum in the first two sums is identical

and given by

$$p_k^{DH} = p_k^{HD} = \frac{J_e^2}{2\left(J_e^2 + M - 2\right)} \frac{\binom{R-1}{k}^2}{\binom{2R-2}{R-1}}. \tag{S79}$$

Similarly, the third and fourth sums have the same coefficients given by

$$p_k^1 = p_k^2 = \frac{(R-1)}{(J_e^2 + M - 2)} \frac{\binom{R-2}{k}\binom{R}{k+1}}{\binom{2R-2}{R-1}}. \tag{S80}$$

The maximal entanglement entropy is obtained for $J_e = J_e^\star$ and scales as $\sqrt{M}$. In that case, we find numerically that $S_{2,\perp}^{\max} = S_{1,\perp} + \log 4$ in the large-M limit. It is easy to understand how this additive factor can appear. For the first family of scarred states, we have $R+1$ non-zero values in the entanglement spectrum, while in this case, we have $4R-2$. For R very large, this is a fourfold increase in the number of non-zero values. As they have a similar distribution, this leads to a simple additive factor of 4 due to the log involved in the calculation.

The case with minimal entanglement entropy is in the limit of a single $J_{e,k}$ (not the middle) being much larger than all other ones. For simplicity, let us consider $J_{e,k} = \delta_{1,k}$. Then the state can be decomposed as

$$\begin{aligned} |E'_R\rangle = \frac{1}{\sqrt{2\binom{2R-2}{R-1}}} \sum_{k=0}^{R-2} \Big[\left(|\mathbf{DH}\rangle + |\mathbf{HD}\rangle\right) \otimes |R{-}2{-}k, k\rangle \Big] \\ \otimes |k{+}1, R{-}1{-}k\rangle \,. \end{aligned} \tag{S81}$$

This gives us only $R-1$ Schmidt values and the entanglement spectrum can be written down as

$$p_k = \frac{\binom{R-2}{k}\binom{R}{k+1}}{\binom{2R-2}{R-1}}. \tag{S82}$$

In the limit of large M, we recover the same result as for the scarred state of the first family $S_{2,\perp}^{\min} = S_{1,\perp} = 0.5 + 0.5\log(\pi M/8)$. This can, once again, be understood simply from the number of nonzero values in the entanglement spectrum as their distribution is similar in both cases. As they have, respectively, $R-1$ and $R+1$ such values, they become identical at the leading order in the large M limit.

For all three cases, we can compute the entanglement entropy efficiently from the analytical form of the entanglement spectrum for systems with hundreds of sites. Fig. S7 displays this along with the expected large-M behavior. We find a very good agreement between them already at $M \approx 20$, with the difference between them decreasing as $1/M$.

While we do not have proof that the cases treated are the true maximum and minimum of $S_{2,\perp}$ we now illustrate numerically that they provide excellent bounds in the large N limit. Fig. S8 shows the entanglement entropy obtained for multiple random realizations with different ranges of parameters and for numerical minimization and maximization over all $J_{e,k}$ and ω_k parameters. For the minimum, we find exact agreement between our analytical and numerical results. For the maximum case, we find that in smaller systems, realizations with high disorder can have higher entanglement entropy than our analytical Ansatz. Nonetheless, the difference between the numerical and analytical maxima converges as N gets larger. This is confirmed by looking at the entanglement spectrum. In all states obtained by numerical maximization of entropy, there are exactly $N = 4R$ nonzero values in it. This precludes them from being volume-law states. Asymptotically, this will also be equivalent to the $4R - 2$ nonzero values in our analytical Ansatz, up to $1/N$ corrections. For these reasons, we believe that the scarred states of the second family cannot be volume-law entangled and that our analytical Ansatz provides an upper-bound in the large N limit.

The maxima of entanglement entropy obtained numerically are also useful to show that it cannot only be reached by fine-tuned cases where many parameters are identical. As these cases might be integrable or have additional symmetries, they are usually not chaotic. Meanwhile, the numerical maxima do not have degenerate parameters and generically show characteristics of ergodic systems. This can be seen for example in the $N = 12$ case, for which the entanglement entropy of eigenstates is plotted in Fig. S8. The concentration of points around an arc is typical of systems obeying the ETH.

10 General symmetry sectors

We have studied different sectors of $\hat{Q}$ symmetry at non-zero magnetization, i.e., at general filling factors in the fermion representation of the model. Fig. S9 shows the entanglement entropy of eigenstates at half-filling (zero magnetization). This is similar to the data presented in the main text, but we plot individual $\hat{Q}$ sectors for increased clarity. The scarred states are clearly visible, while the rest of the eigenstates show a narrow arc-like distribution, typical of a chaotic system. By contrast, Fig. S10 shows a sector away from half-filling, where the same arc is visible. However, in this case, no scarred state is present. Indeed, scarred eigenstates only exist at half-filling, and this can be directly understood from the rainbow scar construction. As the mirror transformation between the two subsystems $\mathcal{M} = \left(\prod_{k=1}^{M} \hat{\mathbf{d}}_k^x\right) \hat{\mathcal{P}}_{\mathbf{d}\leftrightarrow\mathbf{u}}$ involves a particle-hole exchange, the number of particles in subsystems 1 and 2 must sum to M. So by construction, rainbow scars in our model can only exist at half-filling.

11 Generalization to a non-integrable subsystem Hamiltonian

In this section we demonstrate that our results can be generalized to the case in which the subsystem Hamiltonian $\hat{H}_1$ defines a *non-integrable* model. The model presented below is a disordered XY chain with nearest-neighbor and next-nearest-neighbor couplings along the x

and y directions. We use the same notation as in the main text and define

$$
\begin{aligned}
\hat{H} = & \sum_{j=1}^{M-1} J^1_{x,j} \hat{\mathbf{u}}^x_j \hat{\mathbf{u}}^x_{j+1} + J^1_{y,j} \hat{\mathbf{u}}^y_j \hat{\mathbf{u}}^y_{j+1} \\
& + \sum_{j=1}^{M-2} J^2_{x,j} \hat{\mathbf{u}}^x_j \hat{\mathbf{u}}^x_{j+2} + J^2_{y,j} \hat{\mathbf{u}}^y_j \hat{\mathbf{u}}^y_{j+2} \\
& - \sum_{j=1}^{M-1} J^1_{x,j} \hat{\mathbf{d}}^x_j \hat{\mathbf{d}}^x_{j+1} + J^1_{y,j} \hat{\mathbf{d}}^y_j \hat{\mathbf{d}}^y_{j+1} \\
& - \sum_{j=1}^{M-2} J^2_{x,j} \hat{\mathbf{d}}^x_j \hat{\mathbf{d}}^x_{j+2} + J^2_{y,j} \hat{\mathbf{d}}^y_j \hat{\mathbf{d}}^y_{j+2} \\
& + \frac{1}{2} \sum_{j=1}^{M} \hat{\mathbf{u}}^x_j \hat{\mathbf{d}}^x_j + \hat{\mathbf{u}}^y_j \hat{\mathbf{d}}^y_j .
\end{aligned}
\tag{S83}
$$

This Hamiltonian also naturally decomposes into three parts: $\hat{H}_1$ (first two lines) acting on the top row of the ladder, $\hat{H}_2$ (third and fourth line) acting on the bottom row, and $\hat{H}_{\text{int}}$ linking the two. We can see directly that $\hat{H}_2 = -\hat{H}_1$ as the mirror transformation simply swaps the top and bottom row.

The Hamiltonian $\hat{H}_1$ has some symmetries linked to π pulses along the x or z axes. For an even length M, we have two operators $\hat{Z}_x = \prod_{j=1}^{M} \hat{\mathbf{u}}^x_j$ and $\hat{Z}_z = \prod_{j=1}^{M} \hat{\mathbf{u}}^z_j$. Once these symmetries have been resolved, the system is clearly chaotic, as is shown in Fig. S11.

We can now build the rainbow scar state starting from

$$
|\mathbf{I}\rangle = \frac{1}{2^{M/2}} \bigotimes_{k=1}^{M} \left(|{\substack{\circ \\ \circ}}\rangle + |{\substack{\bullet \\ \bullet}}\rangle \right) . \tag{S84}
$$

By acting with $\hat{Z}_x$ and $\hat{Z}_z$ we can generate two more rainbow scars as

$$
|\mathbf{I}_T\rangle = \frac{1}{2^{M/2}} \bigotimes_{k=1}^{M} \left(|{\substack{\circ \\ \bullet}}\rangle + |{\substack{\bullet \\ \circ}}\rangle \right) = |\mathbf{TT} \ldots \mathbf{T}\rangle \tag{S85}
$$

$$
|\mathbf{I}_S\rangle = \frac{1}{2^{M/2}} \bigotimes_{k=1}^{M} \left(|{\substack{\circ \\ \bullet}}\rangle - |{\substack{\bullet \\ \circ}}\rangle \right) = |\mathbf{SS} \ldots \mathbf{S}\rangle . \tag{S86}
$$

These three states have energy respectively 0, M, and $-M$, and have no entanglement entropy for a cut perpendicular to the ladder. We can finally create disordered scarred states (scarred states of the second family) by acting with $\hat{H}_1$ on $|\mathbf{I}_T\rangle$ and $|\mathbf{I}_S\rangle$ to get $|\mathbf{I}_T^2\rangle \propto \hat{H}_1 |\mathbf{I}_T\rangle$ and $|\mathbf{I}_S^2\rangle \propto \hat{H}_1 |\mathbf{I}_S\rangle$ which have energy $M-2$ and $2-M$. These states now have doublon-hole pairs $\mathbf{HD}$ and $\mathbf{DH}$ as well as such pairs with a triplet/singlet in between $\mathbf{HSD}$, $\mathbf{HTD}$, $\mathbf{DTH}$ and $\mathbf{DSH}$. The weights of the configuration depend on their location in the ladder and are directly related to the Hamiltonian parameters $J^i_{\alpha,k}$. Similar to the model studied in the main text, these are entangled states with weights that can be tuned. The entropy of the eigenstates is shown in Fig. S12 This example is simpler as the symmetries of $\hat{H}_1$ do not appear in $\hat{H}_1$ itself. The Hamiltonian $\hat{H}_1$ is also clearly not integrable, showing that integrability is by no means necessary to build disordered scarred states.

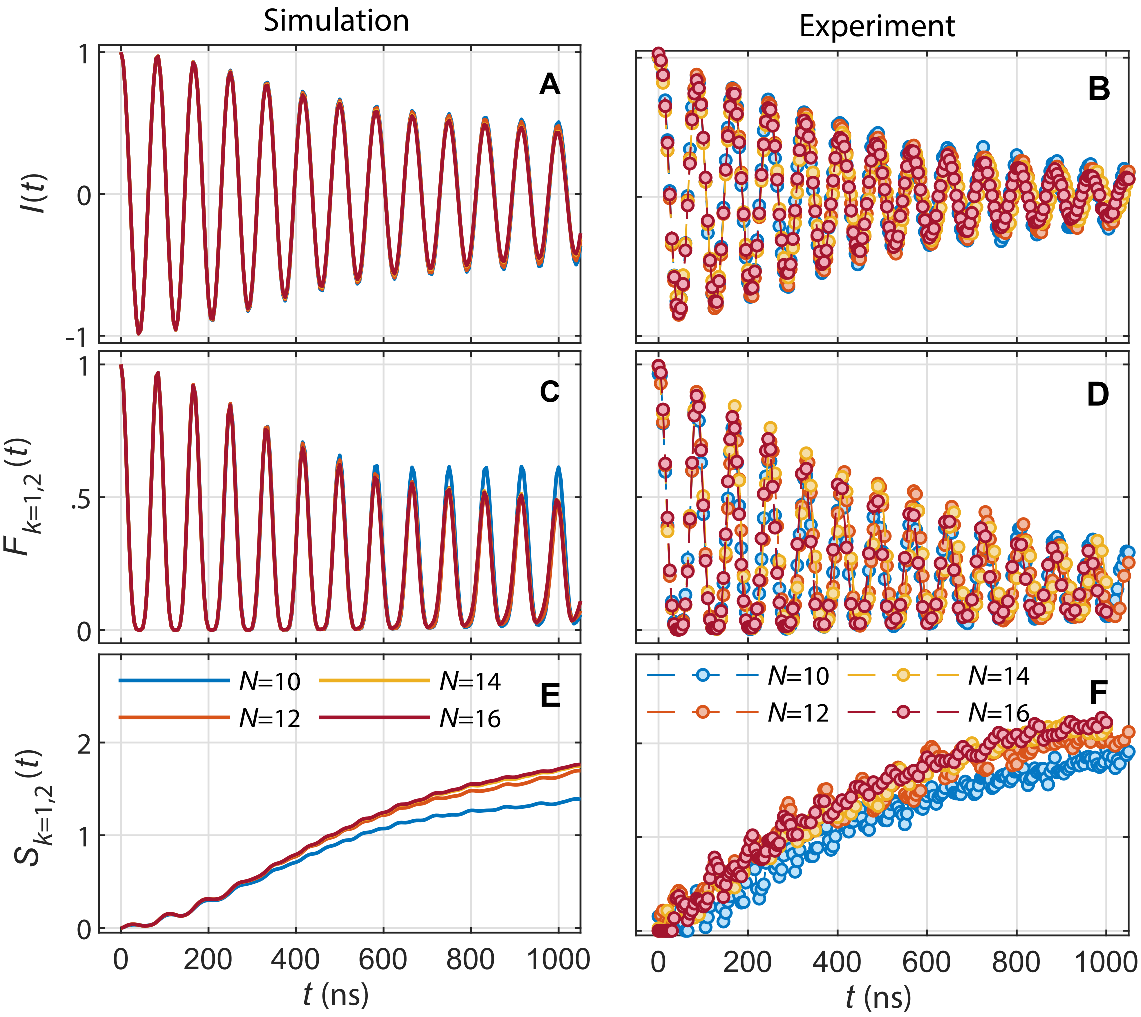

Figure S1: **System size scaling of dynamics.** Dynamics of imbalance (**A**-**B**), subsystem fidelity (**C**-**D**), and entanglement entropy (**E**-**F**) for simulations and experiments, respectively, with different system sizes ranging from $N = 10$ to 16. Data is for the first type of scar, with the same parameters as those used in Fig. 3 of the main text.

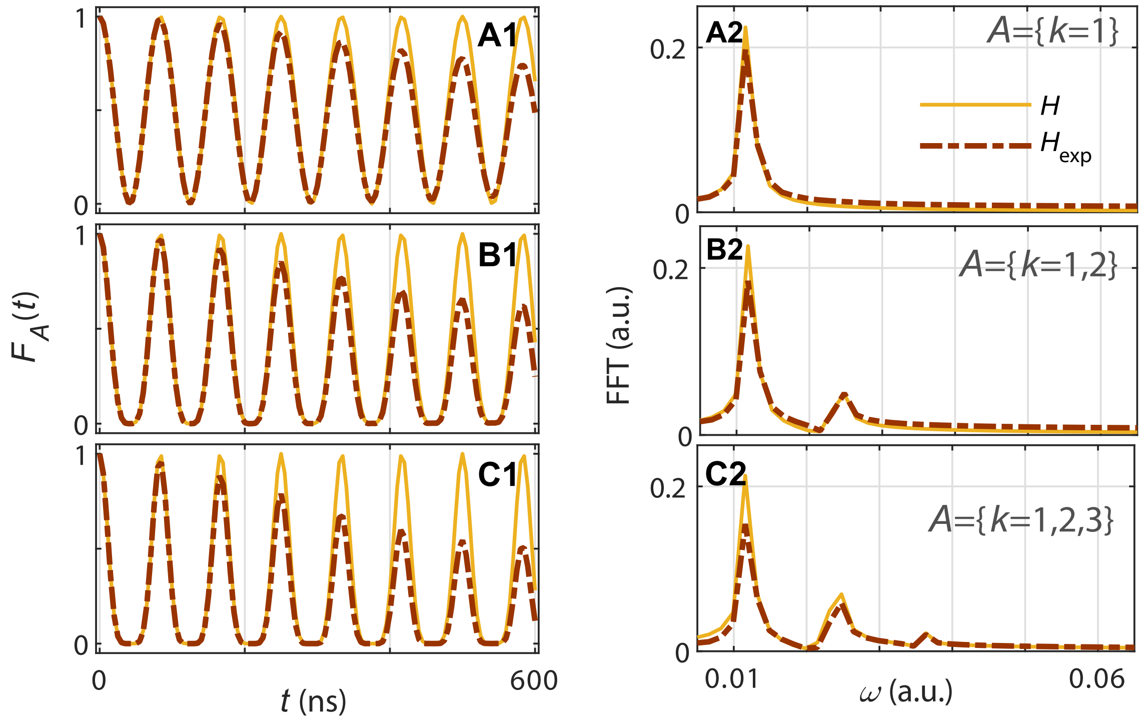

Figure S2: **Scaling of fidelity with subsystem size.** Subsystem fidelity dynamics of the initial state $|\Pi\rangle$ for Hamiltonians $\hat{H}$ in the main text and $\hat{H}_{\rm exp}$ in Eq. (8) for subsystem sizes of $2, 4, 6$, respectively. System size is $N = 10$ and the coupling parameters are $J_a = J_e$, $J_x = 0.1J_a$.

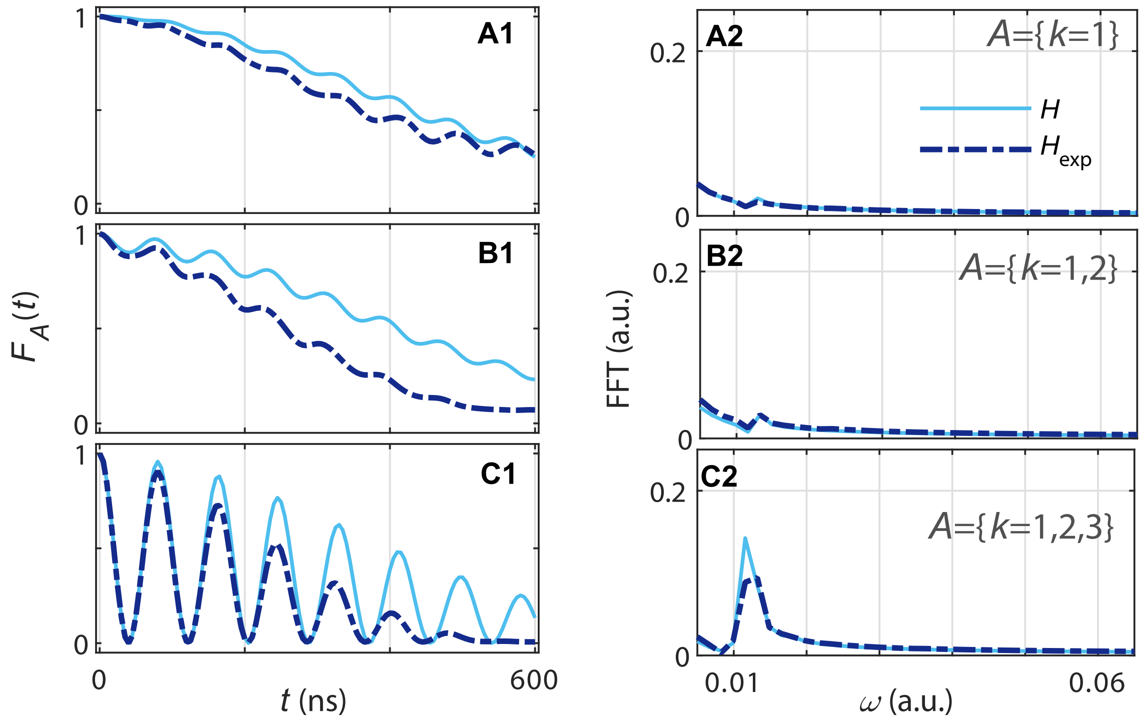

Figure S3: **Fidelity dynamics.** Subsystem fidelity dynamics of the initial state $|\phi_L\rangle$ for the Hamiltonian $\hat{H}$ in the main text and $\hat{H}_{\text{exp}}$ in Eq. (8) of the main text for subsystem sizes of $2, 4, 6$. The parameters are identical to those in Fig. S2.

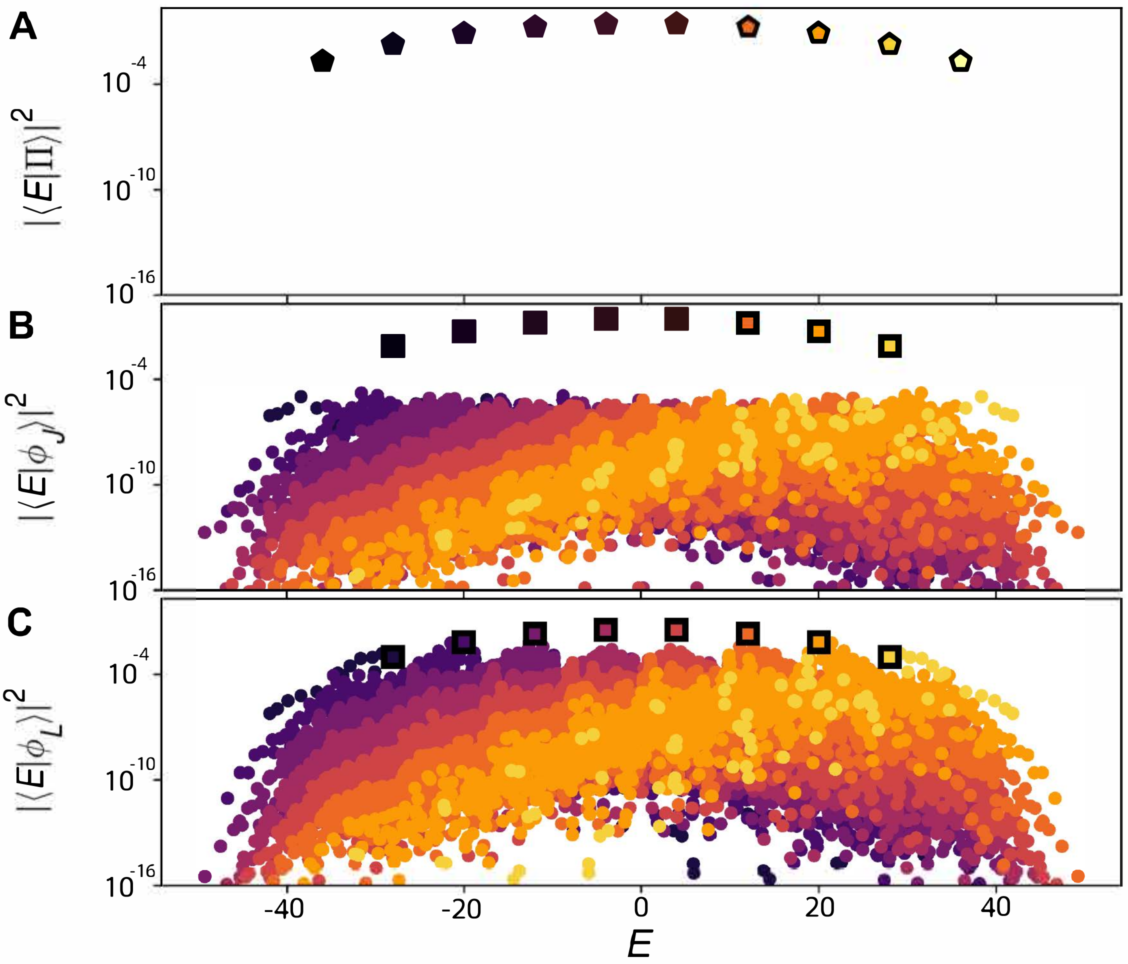

Figure S4: **Support of special initial states.** Overlap between the $|\mathrm{II}\rangle$, $|\phi_J\rangle$ and $|\phi_L\rangle$ states and the eigenstates of the model in Eq. (1). Scarred states of the first family are denoted by pentagons while those of the second family are denoted by squares. $|\mathrm{II}\rangle$ only has overlap on the scarred eigenstates of the first family, while $|\phi_J\rangle$ and $|\phi_L\rangle$ have no overlap on them. For both of these states, the scarred states of the second family dominate. Data is for $N = 18$, $J_a = 4$ and $J_{e,k} \in [4, 4.5], \omega_k \in [0.5, 1.5]$ drawn from a uniform distribution.

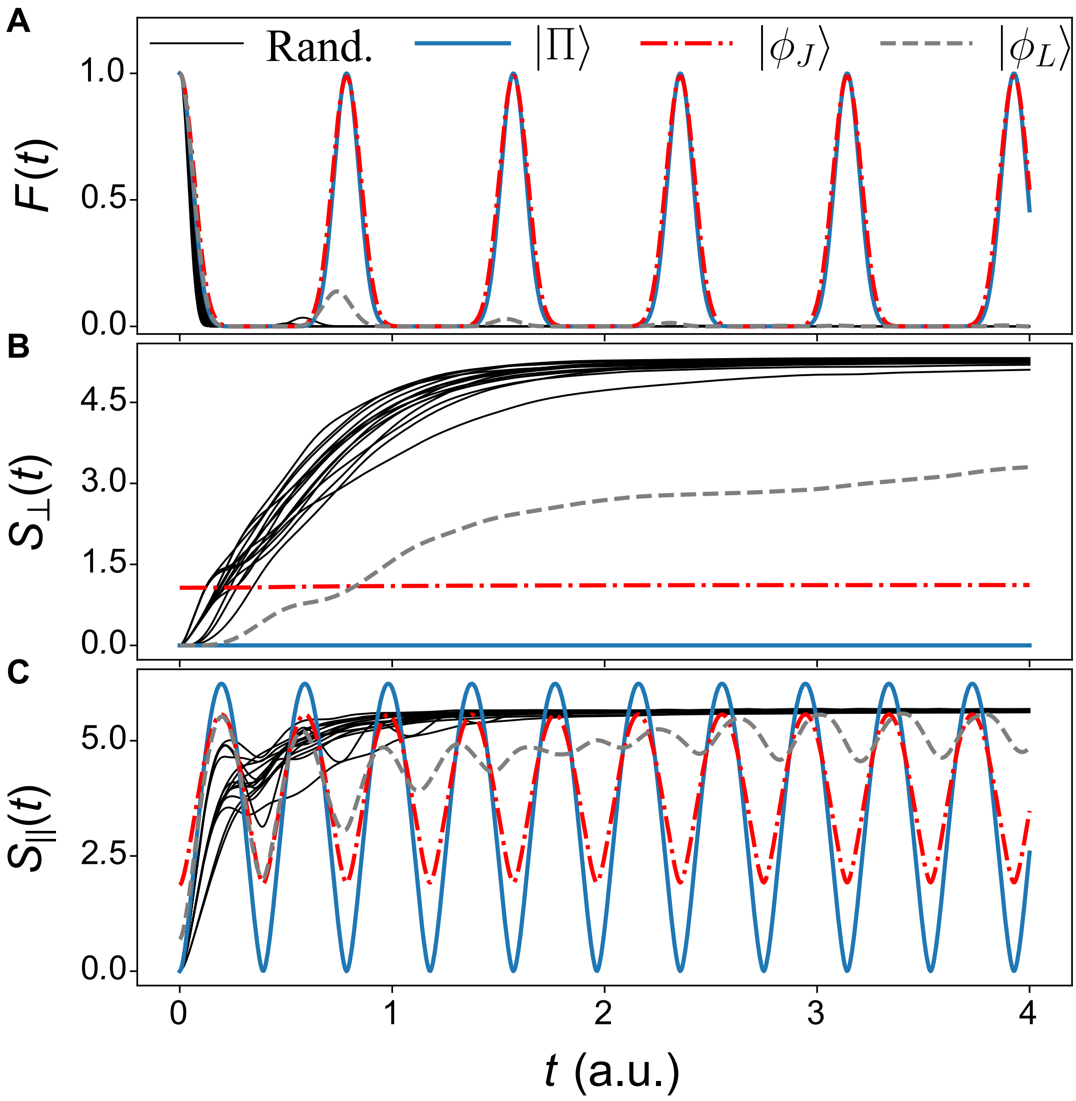

Figure S5: **Dynamics from special initial states.** Fidelity and bipartite entanglement entropy over time following the quench from various initial states indicated in the legend. The thin black lines are randomly chosen Fock basis states at half filling. For $|\Pi\rangle$ and $|\phi_J\rangle$ there is no visible growth of entanglement entropy, while for $|\phi_L\rangle$ the growth is strongly suppressed. Data is for $N = 18$, $J_a = 4$ and $J_{e,k} \in [4, 4.5]$, $\omega_k \in [0.5, 1.5]$ drawn from a uniform distribution.

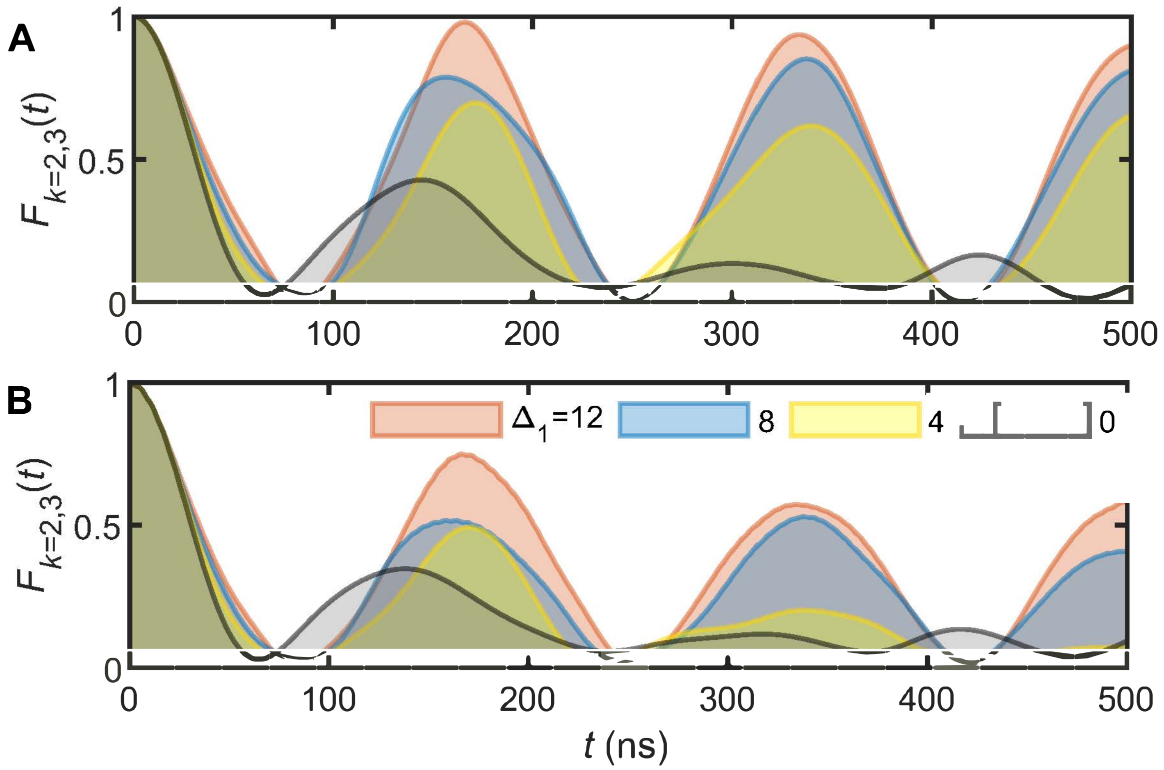

Figure S6: **Tuning of revival by disorder.** Improving the revivals by modulating the hopping strength on the first site according to $J_{e,1} = J^0_{e,1} + \Delta_1$ for the ideal model (**A**) and for the model with experimental imperfections (**B**) in numerics. Monotonic increase of revival peaks can be seen as Δ_1 is increased. Data is for $N = 8$ qubits, $J_a = 3.0$ MHz, $J_{e,k} \in [2.0, 3.0]$ MHz, drawn from a uniform distribution.

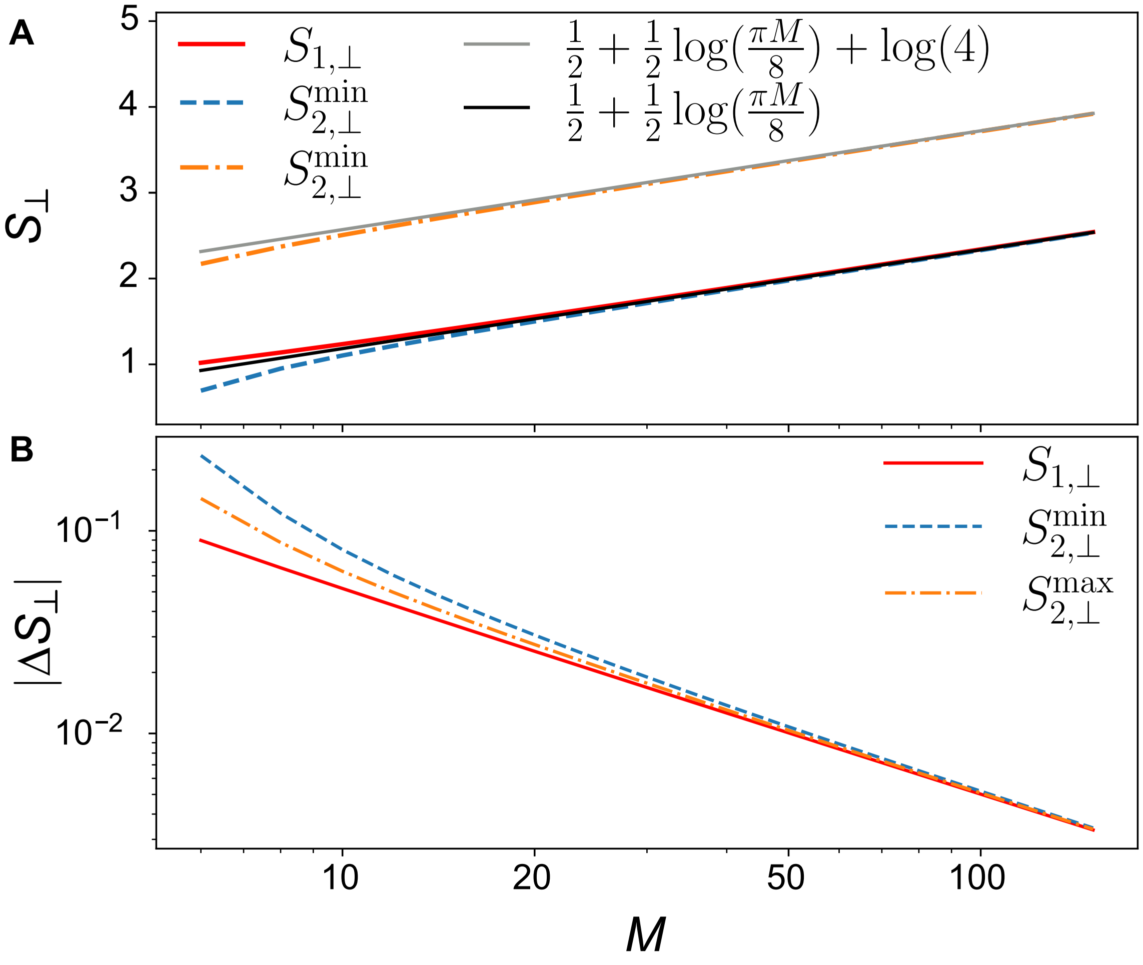

Figure S7: **Asymptotic entanglement entropy.** (**A**) Entanglement entropy computed from the analytical formula of the entanglement spectrum. The gray and black lines represent the expected large-M limit and they are in good agreement with the data already for $N \approx 20$. (**B**) Difference between the data and the large-M prediction. For all three cases, the difference approximately decays as $\propto 1/M$.

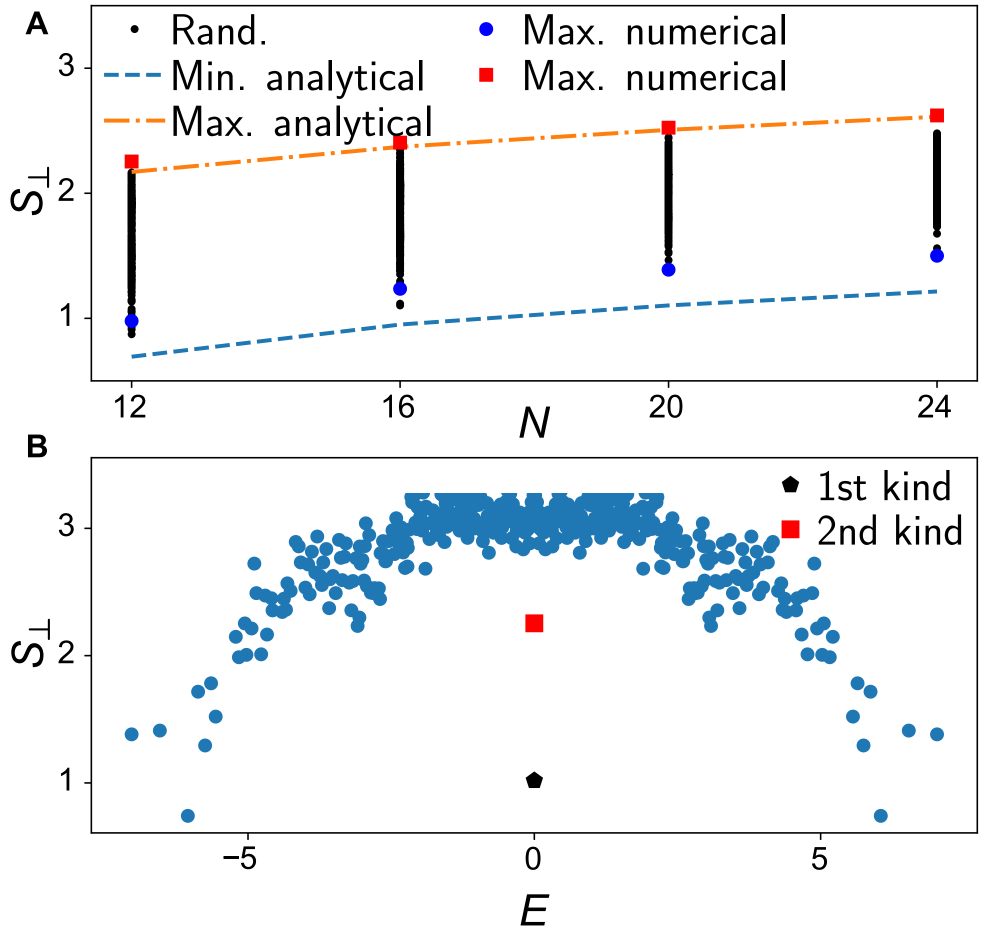

Figure S8: **Entanglement entropy for different disorder realizations.** (**A**) Entanglement entropy of the scarred state of the second family with $E = 0$ for random realizations, for the analytical formulas, and from numerical minimization/maximization. As N increases, the analytical and numerical results converge. (**B**) Entanglement entropy of eigenstates in the $Q = 0$ sector for $N = 12$ in the parameter regime was found to maximize the entanglement entropy of the scarred state of the second family. The non-scarred eigenstates concentrate around an arc, as is typical of chaotic systems. This shows that high entanglement entropy can be obtained without getting close to a fine-tuned integrable point where all parameters are identical.

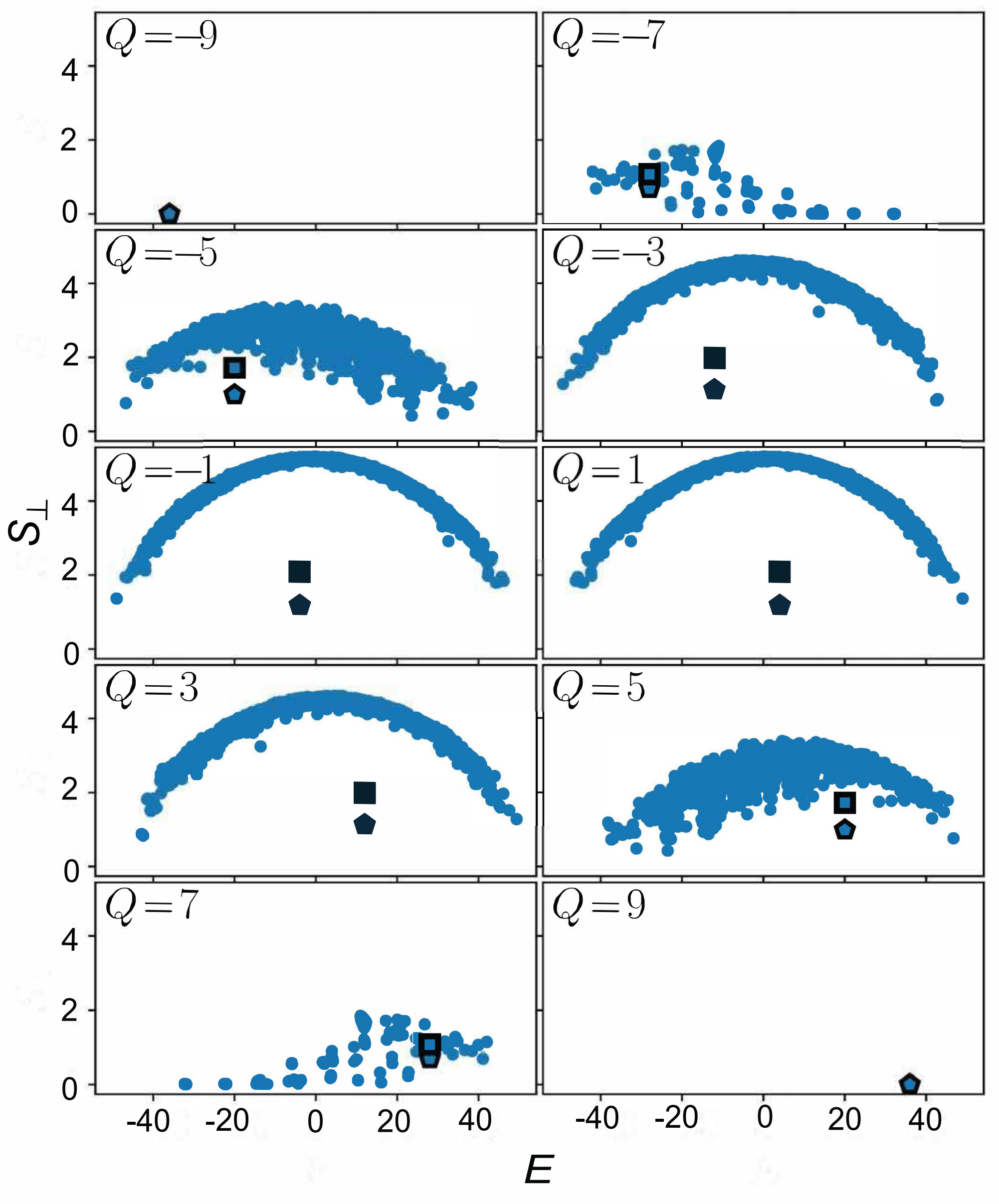

Figure S9: **Entanglement entropy in all symmetry sectors.** Entanglement entropy of the eigenstates for a chain with $N = 18$, $J_a = 4$, $J_{e,k} \in [2, 6]$, and $\omega_k \in [1, 3]$. Each subplot corresponds to a different sector of Q. The scarred states of the first and second families are denoted by pentagons and squares respectively.

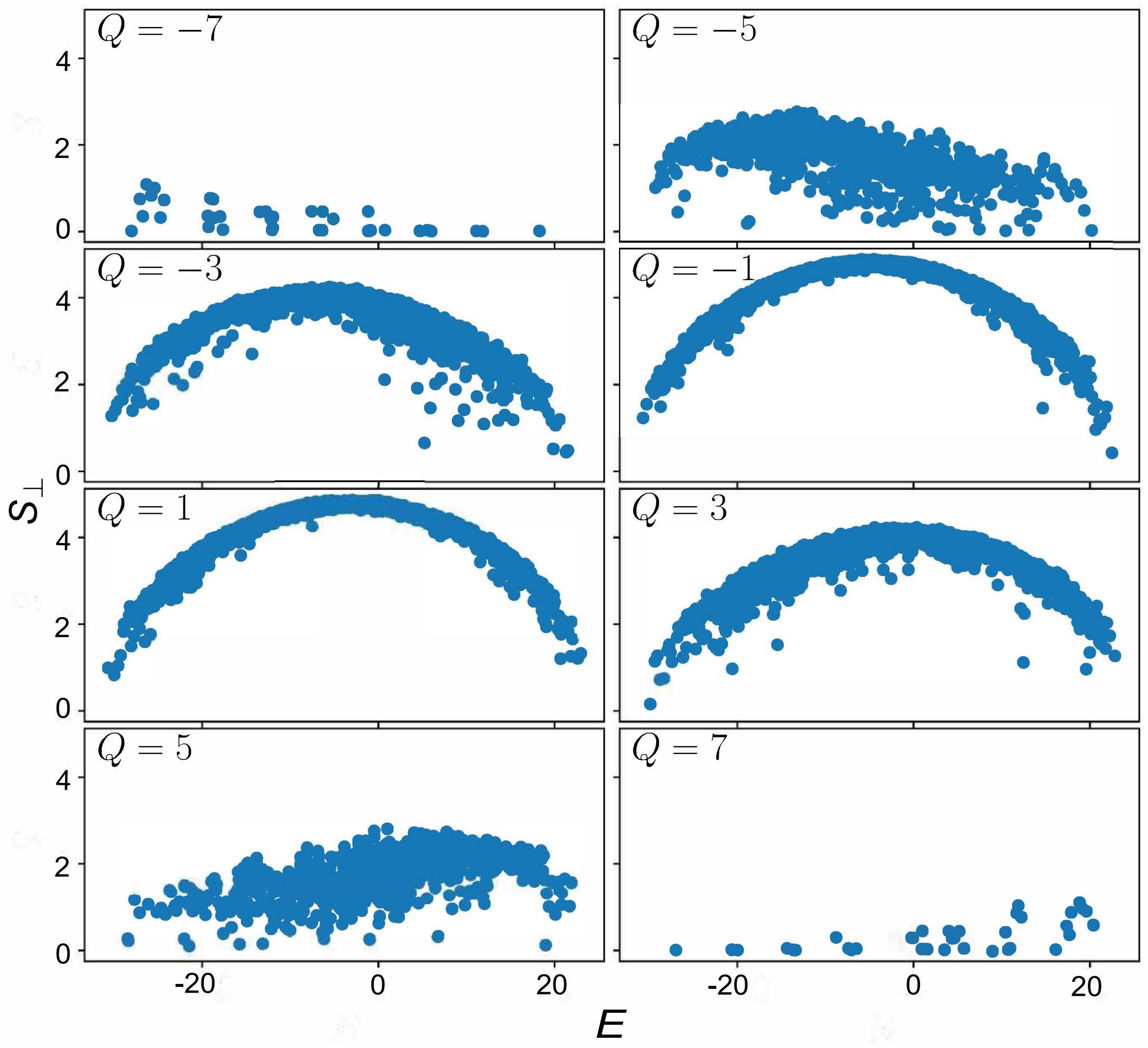

Figure S10: **Entanglement entropy away from half-filling.** Entanglement entropy of the eigenstates for a chain with $N = 18$ and 7 excitations. Unlike at half-filling (9 excitations), no low-entropy scarred eigenstates can be seen. The system parameters are $J_a = 3$, $J_{e,k} \in [2, 2.5]$, and $\omega_k \in [0.5, 1.5]$.

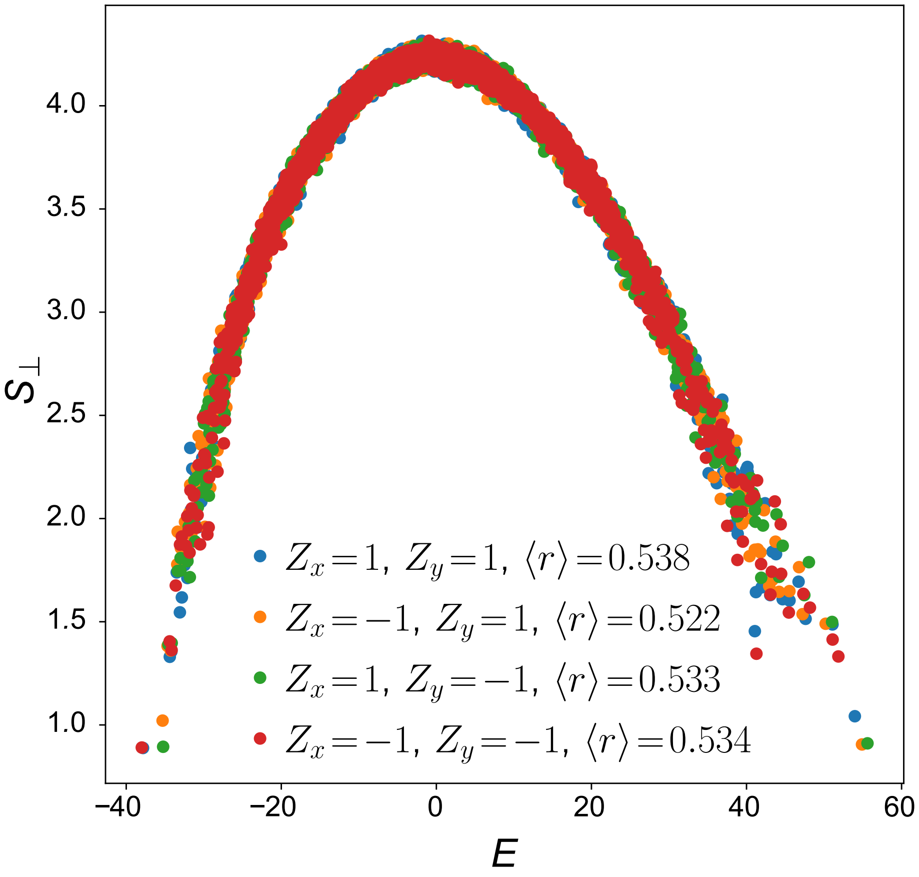

Figure S11: **Entanglement entropy in the range-2 XY chain.** Entanglement entropy of the eigenstates with $M = 14$, $J^1_{x,k} \in [2.3, 2.4]$, $J^1_{y,k} \in [1.2, 1.4]$, $J^2_{x,k} = [1.3, 1.4]$, $J^2_{y,k} l \in [2.5, 2.7]$. Once the symmetries have been resolved, the level statistics indicate that the system is chaotic (see legend).

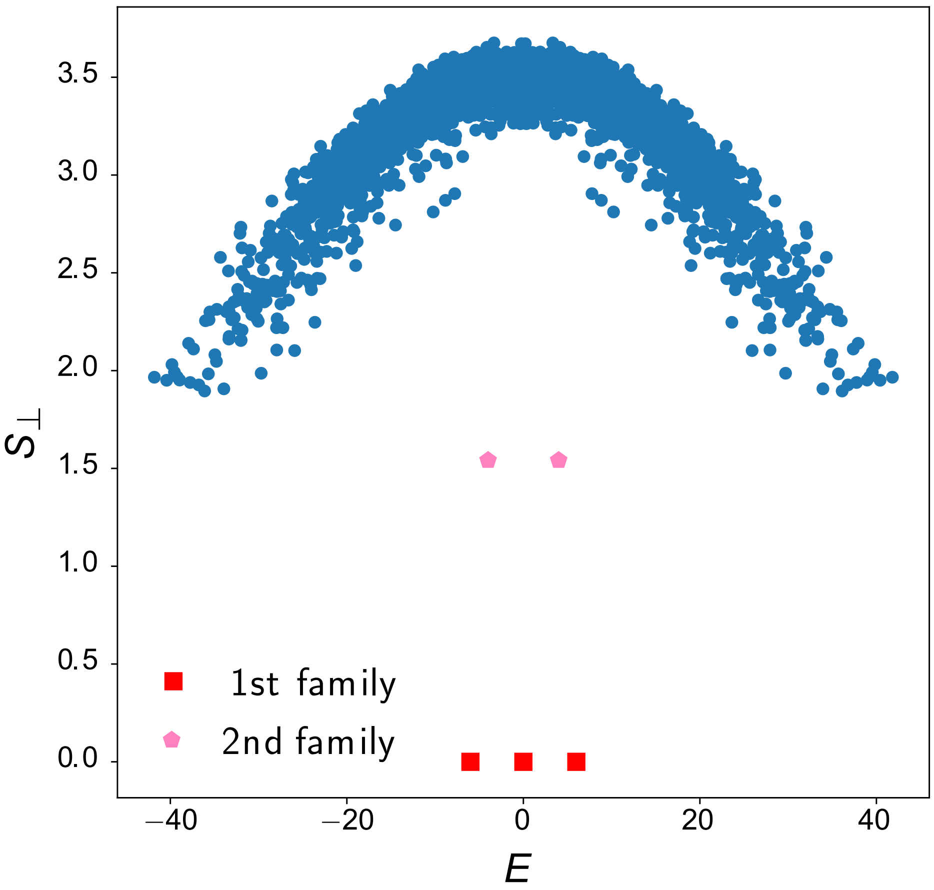

Figure S12: **Entanglement entropy in the range-2 XY ladder.** Entanglement entropy of the eigenstates with $N = 12$, $J^1_{x,k} \in [2.3, 2.4]$, $J^1_{y,k} \in [1.2, 1.4]$, $J^2_{x,k} = [1.9, 2]$, $J^2_{y,k} l \in [3.1, 3.3]$. The two types of scarred states are well separated in entropy and are clearly distinguishable from the rest of the spectrum.

REFERENCES AND NOTES

1. J. M. Deutsch, Quantum statistical mechanics in a closed system, *Phys. Rev. A* **43**, 2046–2049 (1991).

2. M. Srednicki, Chaos and quantum thermalization, *Phys. Rev. E.* **50**, 888–901 (1994).

3. M. Rigol, V. Dunjko, M. Olshanii, Thermalization and its mechanism for generic isolated quantum systems, *Nature* **452**, 854–858 (2008).

4. T. Kinoshita, T. Wenger, D. S. Weiss, A quantum Newton's cradle, *Nature* **440**, 900–903 (2006).

5. B. Sutherland, *Beautiful Models: 70 Years of Exactly Solved Quantum Many-body Problems* (World Scientific, 2004).

6. P. W. Anderson, Absence of diffusion in certain random lattices, *Phys. Rev.* **109**, 1492–1505 (1958).

7. R. Nandkishore, D. A. Huse, Many-body localization and thermalization in quantum statistical mechanics, *Annu. Rev. Condens. Matter. Phys.* **6**, 15–38 (2015).

8. D. A. Abanin, E. Altman, I. Bloch, M. Serbyn, Colloquium: Many-body localization, thermalization, and entanglement, *Rev. Mod. Phys.* **91**, 021001 (2019).

9. D. A. Huse, R. Nandkishore, V. Oganesyan, A. Pal, S. L. Sondhi, Localization-protected quantum order, *Phys. Rev. B* **88**, 014206 (2013).

10. Y. Bahri, R. Vosk, E. Altman, A. Vishwanath, Localization and topology protected quantum coherence at the edge of hot matter *Nat. Commun.* **6**, 7341 (2015).

11. B. Bauer, C. Nayak, Area laws in a many-body localized state and its implications for topological order, *J. Stat. Mech. Theory Exp.* **2013**, P09005 (2013).

12. L. Pezzè, A. Smerzi, M. K. Oberthaler, R. Schmied, P. Treutlein, Quantum metrology with nonclassical states of atomic ensembles, *Rev. Mod. Phys.* **90**, 035005 (2018).

13. J.-Y. Desaules, F. Pietracaprina, Z. Papić, J. Goold, S. Pappalardi, Extensive multipartite entanglement from su(2) quantum many-body scars, *Phys. Rev. Lett.* **129**, 020601 (2022).

14. S. Dooley, S. Pappalardi, J. Goold, Entanglement enhanced metrology with quantum many-body scars, *Phys. Rev. B* **107**, 035123 (2023).

15. M. Serbyn, D. A. Abanin, Z. Papić, Quantum many-body scars and weak breaking of ergodicity, *Nat. Phys.* **17**, 675–685 (2021).

16. S. Moudgalya, B. A. Bernevig, N. Regnault, Quantum many-body scars and Hilbert space fragmentation: A review of exact results, *Rep. Prog. Phys.* **85**, 086501 (2022).

17. A. Chandran, T. Iadecola, V. Khemani, R. Moessner, Quantum many-body scars: A quasiparticle perspective, *Annu. Rev. Condens. Matter. Phys.* **14**, 443–469 (2023).

18. H. Bernien, S. Schwartz, A. Keesling, H. Levine, A. Omran, H. Pichler, S. Choi, A. S. Zibrov, M. Endres, M. Greiner, V. Vuletic, M. D. Lukin, Probing many-body dynamics on a 51-atom quantum simulator, *Nature* **551**, 579 (2017), 584.

19. D. Bluvstein, A. Omran, H. Levine, A. Keesling, G. Semeghini, S. Ebadi, T. T. Wang, A. A. Michailidis, N. Maskara, W. W. Ho, S. Choi, M. Serbyn, M. Greiner, V. Vuletić, M. D. Lukin, Controlling quantum many-body dynamics in driven Rydberg atom arrays, *Science* **371**, 1355–1359 (2021).

20. P. N. Jepsen, Y. K. E. Lee, H. Lin, I. Dimitrova, Y. Margalit, W. W. Ho, W. Ketterle, Long-lived phantom helix states in Heisenberg quantum magnets, *Nat. Phys.* **18**, 899–904 (2022).

21. G.-X. Su, H. Sun, A. Hudomal, J.-Y. Desaules, Z.-Y. Zhou, B. Yang, J. C. Halimeh, Z.-S. Yuan, Z. Papić, J.-W. Pan, Observation of many-body scarring in a Bose-Hubbard quantum simulator, *Phys. Rev. Res.* **5**, 023010 (2023).

22. P. Zhang, H. Dong, Y. Gao, L. Zhao, J. Hao, J.-Y. Desaules, Q. Guo, J. Chen, J. Deng, B. Liu, W. Ren, Y. Yao, X. Zhang, S. Xu, K. Wang, F. Jin, X. Zhu, B. Zhang, H. Li, C. Song, Z. Wang, F. Liu, Z. Papić, L. Ying, H. Wang, Y.-C. Lai, Many-body Hilbert space scarring on a superconducting processor, *Nat. Phys.* **19**, 120–125 (2023).

23. Y. Yao, L. Xiang, Z. Guo, Z. Bao, Y.-F. Yang, Z. Song, H. Shi, X. Zhu, F. Jin, J. Chen, S. Xu, Z. Zhu, F. Shen, N. Wang, C. Zhang, Y. Wu, Y. Zou, P. Zhang, H. Li, Z. Wang, C. Song, C. Cheng, R. Mondaini, H. Wang, J. Q. You, S.-Y. Zhu, L. Ying, Q. Guo, Observation of many-body Fock space dynamics in two dimensions, *Nat. Phys.* **19**, 1459–1465 (2023).

24. N. Shiraishi, T. Mori, Systematic construction of counterexamples to the Eigenstate thermalization hypothesis, *Phys. Rev. Lett.* **119**, 030601 (2017).

25. S. Moudgalya, N. Regnault, B. A. Bernevig, Entanglement of exact excited states of Affleck-Kennedy-Lieb-Tasaki models: Exact results, many-body scars, and violation of the strong Eigenstate thermalization hypothesis, *Phys. Rev. B* **98**, 235156 (2018).

26. C. J. Turner, A. A. Michailidis, D. A. Abanin, M. Serbyn, Z. Papić, Weak ergodicity breaking from quantum many-body scars, *Nat. Phys.* **14**, 745–749 (2018).

27. W. W. Ho, S. Choi, H. Pichler, M. D. Lukin, Periodic orbits, entanglement, and quantum many-body scars in constrained models: Matrix product state approach, *Phys. Rev. Lett.* **122**, 040603 (2019).

28. M. Schecter, T. Iadecola, Weak ergodicity breaking and quantum many-body scars in spin-1 XY magnets, *Phys. Rev. Lett.* **123**, 147201 (2019).

29. D. K. Mark, C.-J. Lin, O. I. Motrunich, Unified structure for exact towers of scar states in the Affleck-Kennedy-Lieb-Tasaki and other models, *Phys. Rev. B* **101**, 195131 (2020).

30. N. O'Dea, F. Burnell, A. Chandran, V. Khemani, From tunnels to towers: Quantum scars from Lie algebras and q-deformed Lie algebras, *Phys. Rev. Research* **2**, 043305 (2020).

31. K. Pakrouski, P. N. Pallegar, F. K. Popov, I. R. Klebanov, Many-body scars as a group invariant sector of Hilbert space, *Phys. Rev. Lett.* **125**, 230602 (2020).

32. S. Moudgalya, O. I. Motrunich, Exhaustive characterization of quantum many-body scars using commutant algebras. arXiv:2209.03377 [cond-mat.str-el] (2022).

33. B. Buča, Unified theory of local quantum many-body dynamics: Eigenoperator thermalization theorems, *Phys. Rev. X* **13**, 031013 (2023).

34. C. M. Langlett, Z.-C. Yang, J. Wildeboer, A. V. Gorshkov, T. Iadecola, S. Xu, Rainbow scars: From area to volume law, *Phys. Rev. B* **105**, L060301 (2022).

35. J. Wildeboer, C. M. Langlett, Z.-C. Yang, A. V. Gorshkov, T. Iadecola, S. Xu, Quantum many-body scars from Einstein-Podolsky-Rosen states in bilayer systems, *Phys. Rev. B* **106**, 205142 (2022).

36. N. Shibata, N. Yoshioka, H. Katsura, Onsager's scars in disordered spin chains, *Phys. Rev. Lett.* **124**, 180604 (2020).

37. I. Mondragon-Shem, M. G. Vavilov, I. Martin, Fate of quantum many-body scars in the presence of disorder, *PRX Quantum* **2**, 030349 (2021).

38. K. Huang, Y. Wang, X. Li, Stability of scar states in the two-dimensional PXP model against random disorder, *Phys. Rev. B* **104**, 214305 (2021).

39. B. van Voorden, M. Marcuzzi, K. Schoutens, J. Minář, Disorder enhanced quantum many-body scars in Hilbert hypercubes, *Phys. Rev. B* **103**, L220301 (2021).

40. G. Zhang, Z. Song, Quantum scars in spin- 1/2 isotropic Heisenberg clusters, *New J. Phys.* **25**, 053025 (2023).

41. N. S. Srivatsa, H. Yarloo, R. Moessner, A. E. B. Nielsen, Mobility edges through inverted quantum many-body scarring, *Phys. Rev. B* **108**, L100202 (2023).

42. Q. Chen, Z. Zhu, Inverting multiple quantum many-body scars via disorder. arXiv:2301.03405 [cond-mat.dis-nn] (2023).

43. M. Iversen, A. E. B. Nielsen, Tower of quantum scars in a partially many-body localized system, *Phys. Rev. B* **107**, 205140 (2023).

44. C.-J. Lin, O. I. Motrunich, Exact quantum many-body scar states in the Rydberg-blockaded atom chain, *Phys. Rev. Lett.* **122**, 173401 (2019).

45. F. Arute, K. Arya, R. Babbush, D. Bacon, J. C. Bardin, R. Barends, R. Biswas, S. Boixo, F. G. S. L. Brandao, D. A. Buell, B. Burkett, Y. Chen, Z. Chen, B. Chiaro, R. Collins, W. Courtney, A. Dunsworth, E. Farhi, B. Foxen, A. Fowler, C. Gidney, M. Giustina, R. Graff, K. Guerin, S. Habegger, M. P. Harrigan, M. J. Hartmann, A. Ho, M. Hoffmann, T. Huang, T. S. Humble, S. V. Isakov, E. Jeffrey, Z. Jiang, D. Kafri, K. Kechedzhi, J. Kelly, P. V. Klimov, S. Knysh, A. Korotkov, F. Kostritsa, D. Landhuis, M. Lindmark, E. Lucero, D. Lyakh, S. Mandrà, J. R. Mc Clean, M. M. Ewen, A. Megrant, X. Mi, K. Michielsen, M. Mohseni, J. Mutus, O. Naaman, M. Neeley, C. Neill, M. Y. Niu, E. Ostby, A. Petukhov, J. C. Platt, C. Quintana, E. G. Rieffel, P. Roushan, N. C. Rubin, D. Sank, K. J. Satzinger, V. Smelyanskiy, K. J. Sung, M. D. Trevithick, A. Vainsencher, B. Villalonga, T. White, Z. J. Yao, P. Yeh, A. Zalcman, H. Neven, J. M. Martinis, Quantum supremacy using a programmable superconducting processor, *Nature* **574**, 505–510 (2019).

46. T. Iadecola, M. Žnidarič, Exact localized and ballistic eigenstates in disordered chaotic spin ladders and the Fermi-Hubbard model, *Phys. Rev. Lett.* **123**, 036403 (2019).

47. J. Hauschild, F. Pollmann, Efficient numerical simulations with tensor networks: Tensor network Python (TeNPy). *SciPost Phys. Lect.*, 5 (2018).

48. D. N. Page, Average entropy of a subsystem, *Phys. Rev. Lett.* **71**, 1291–1294 (1993).

49. X. Zhang, W. Jiang, J. Deng, K. Wang, J. Chen, P. Zhang, W. Ren, H. Dong, S. Xu, Y. Gao, F. Jin, X. Zhu, Q. Guo, H. Li, C. Song, A.V. Gorshkov, T. Iadecola, F. Liu, Z.X. Gong, Z. Wang, D.L. Deng, H. Wang , Digital quantum simulation of Floquet symmetry-protected topological phases, *Nature* **607**, 468–473 (2022).
